# RNA binding proteins Smaug and Cup induce CCR4–NOT-dependent deadenylation of the *nanos* mRNA in a reconstituted system

**DOI:** 10.1093/nar/gkad159

**Published:** 2023-03-23

**Authors:** Filip Pekovic, Christiane Rammelt, Jana Kubíková, Jutta Metz, Mandy Jeske, Elmar Wahle

**Affiliations:** Institute of Biochemistry and Biotechnology and Charles Tanford Protein Center, Martin Luther University Halle-Wittenberg, Kurt-Mothes-Strasse 3a, 06120 Halle, Germany; RNA Biology Laboratory, Center for Cancer Research, National Cancer Institute, 1050 Boyles Street, Frederick, MD 21702, USA; Institute of Biochemistry and Biotechnology and Charles Tanford Protein Center, Martin Luther University Halle-Wittenberg, Kurt-Mothes-Strasse 3a, 06120 Halle, Germany; Heidelberg University Biochemistry Center (BZH), Im Neuenheimer Feld 328, 69120 Heidelberg, Germany; Heidelberg University Biochemistry Center (BZH), Im Neuenheimer Feld 328, 69120 Heidelberg, Germany; Heidelberg University Biochemistry Center (BZH), Im Neuenheimer Feld 328, 69120 Heidelberg, Germany; Institute of Biochemistry and Biotechnology and Charles Tanford Protein Center, Martin Luther University Halle-Wittenberg, Kurt-Mothes-Strasse 3a, 06120 Halle, Germany

## Abstract

Posttranscriptional regulation of the maternal *nanos* mRNA is essential for the development of the anterior – posterior axis of the *Drosophila* embryo. The *nanos* RNA is regulated by the protein Smaug, which binds to Smaug recognition elements (SREs) in the *nanos* 3’-UTR and nucleates the assembly of a larger repressor complex including the eIF4E-T paralog Cup and five additional proteins. The Smaug-dependent complex represses translation of *nanos* and induces its deadenylation by the CCR4–NOT deadenylase. Here we report an *in vitro* reconstitution of the *Drosophila* CCR4–NOT complex and Smaug-dependent deadenylation. We find that Smaug by itself is sufficient to cause deadenylation by the *Drosophila* or human CCR4–NOT complexes in an SRE-dependent manner. CCR4–NOT subunits NOT10 and NOT11 are dispensable, but the NOT module, consisting of NOT2, NOT3 and the C-terminal part of NOT1, is required. Smaug interacts with the C-terminal domain of NOT3. Both catalytic subunits of CCR4–NOT contribute to Smaug-dependent deadenylation. Whereas the CCR4–NOT complex itself acts distributively, Smaug induces a processive behavior. The cytoplasmic poly(A) binding protein (PABPC) has a minor inhibitory effect on Smaug-dependent deadenylation. Among the additional constituents of the Smaug-dependent repressor complex, Cup also facilitates CCR4–NOT-dependent deadenylation, both independently and in cooperation with Smaug.

## INTRODUCTION

Poly(A) tails decorate the 3’ ends of almost all eukaryotic mRNAs. Long poly(A) tails are appended to newly made mRNAs in the cell nucleus (1–6) and then gradually shortened by 3’ exonucleases in the cytoplasm (3,7–9). Poly(A) tail shortening (deadenylation) can serve two regulatory purposes: First, deadenylation initiates the decay of most mRNAs and is a prerequisite for their complete degradation, which occurs mostly by hydrolysis of the 5’ cap followed by 5’-3’ degradation (3,10,11). The rates of deadenylation are specific for different mRNAs and are the major determinants of mRNA half-life (3,9,12). Second, deadenylation can contribute to the regulation of translation. Poly(A) tails stimulate the initiation of translation via a protein-mediated interaction with the mRNA 5’ end (13,14). During oocyte maturation and early embryonic development of animals, it is not just the presence but the length of the poly(A) tail that affects the rate of translation, as initially shown by studies of individual mRNAs in several species (15). Transcriptome-wide analyses confirmed a strong correlation between long tails and high translation efficiency at early embryonic stages, but not in non-embryonic cells (16–22). In oocyte maturation and early development, regulated extension or shortening of poly(A) tails is used as a means for translational activation or inactivation, respectively (15,23).

**Figure 1.**
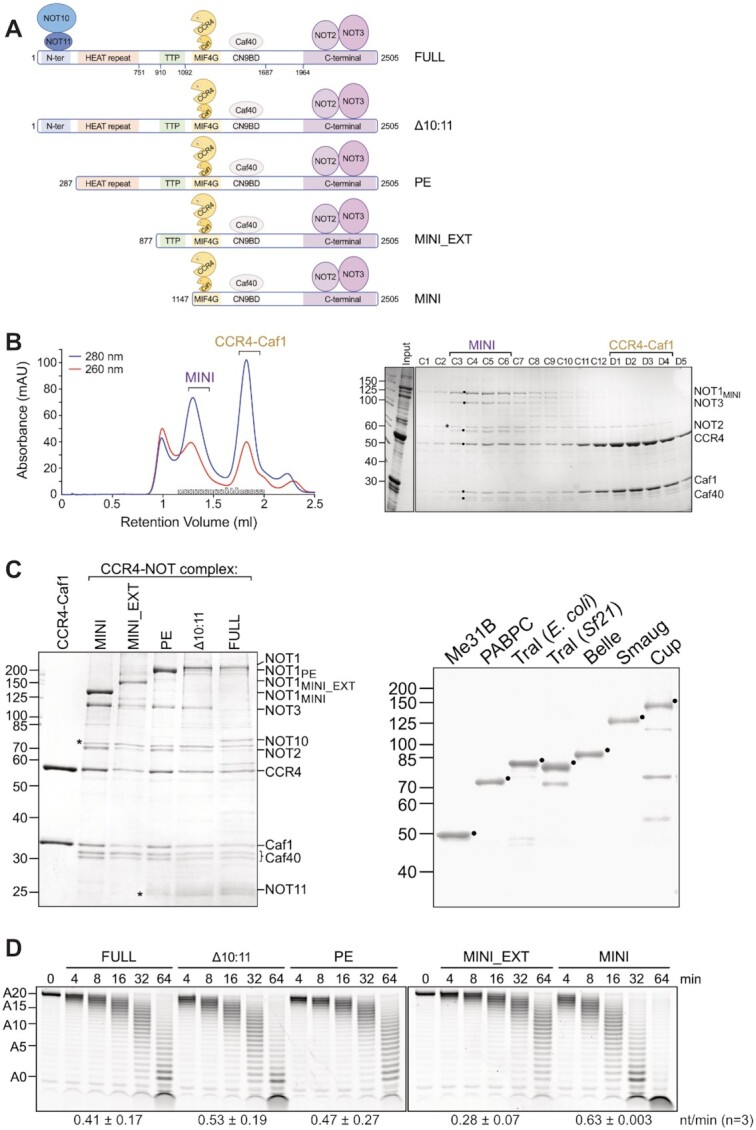
Reconstitution of the *Drosophila melanogaster* CCR4–NOT complex. (**A**) Scheme of different variants of the *Drosophila* CCR4–NOT complex. Domains and interaction sites of NOT1 are denoted as follows: MIF4G, ‘middle of 4G’ domain; CN9BD, CNOT9 (= CAF40) binding domain; TTP denotes the interaction surface. NOT1_PE_ is a naturally occurring isoform. (**B**) Purification of the ^Dm^CCR4–NOT_MINI_ complex by gel filtration. The complex was first purified by FLAG affinity chromatography and then applied to a Superose 6 column as described in Materials and Methods. Left panel, UV profile of the column; right panel, analysis of relevant fractions by SDS polyacrylamide gel electrophoresis and Coomassie staining. Subunits of the CCR4–NOT complex are labeled with black dots and a contaminating band with an asterisk in the gel. (**C**) Purified proteins used in this study. Left panel, preparations of different variants of the ^Dm^CCR4–NOT complex. Prominent contaminants are indicated with asterisks. Right panel, purified components of the SRE-dependent repressor complex. Desired polypeptides are marked. Purified proteins were separated on SDS-polyacrylamide gels and stained with Coomassie. (**D**) Basal activity of different variants of the ^Dm^CCR4–NOT complex. Purified variants of the ^Dm^CCR4–NOT complex (6.25 nM each), were incubated with excess FAM-7mer-A_20_ (50 nM), and aliquots were withdrawn as indicated. Numbers at the bottom represent average deadenylation rates in nt/min plus/minus standard deviation based on *n* = 3.

Cytoplasmic deadenylation of mRNAs is catalyzed mainly by the heterooligomeric CCR4–NOT complex (24–26). The complex is organized around the huge central NOT1 subunit (Figure 1A). The N-terminal region of NOT1 binds two subunits, NOT10 and NOT11, which contribute to substrate RNA recognition and protein-protein interactions (27–30). The MIF4G domain in the middle of NOT1 provides the docking site for the two catalytic subunits: CAF1 (encoded by *Pop2* in *Drosophila*) is bound directly, whereas CCR4 (*twin*) binds CAF1 (31) and thus associates with NOT1 indirectly. CAF40 (*Rcd1*; CNOT9 in humans), which also has affinity for RNA (27,32), associates with the CNOT9 binding domain (CN9BD) of NOT1, which neighbors the MIF4G domain on the C-terminal side (33,34). A NOT2∙NOT3 heterodimer (NOT2 encoded by *Regena*) bound to a C-terminal fragment of NOT1 is termed the NOT module (35,36) (earlier work on the structure of CCR4–NOT reviewed by Wahle and Winkler (7)). The subunits without enzymatic activity not only enhance the activity and substrate specificity of the two exonucleases (27,37,38), but also provide a large surface to interact with numerous effectors of deadenylation. Each effector binds a specific set of mRNAs and promotes their deadenylation. One class of such deadenylation specificity factors are miRNAs, which recruit the CCR4–NOT complex via associated GW182 proteins (39–42). The second class are RNA-binding proteins that, like miRNAs, occupy specific binding sites in 3’ UTRs. Well-characterized examples include tristetraprolin (43–46), Pumilio (44,47–49) and Roquin (50,51), all of which interact directly with the CCR4–NOT complex.

Post-transcriptional regulation, by changes in poly(A) tail length or other means, is essential during early embryonic development of animals: Because the newly formed zygotic genome remains inactive during the first few cell cycles, translation depends on so-called maternal mRNAs. These are synthesized and shelved during oocyte growth and thus contributed to the developing embryo from the maternal genome via the egg (52–54). For example, regulation of the maternal *nanos* (*nos*) mRNA is important for the establishment of the anterior-posterior body axis in *Drosophila*. Nanos protein, the determinant for the formation of posterior structures, is made exclusively by translation of a small fraction of *nos* mRNA that is localized at the posterior pole. In contrast, the larger, non-localized fraction of *nos* mRNA is repressed (55–57). One repressor of *nos* is the protein Smaug, which binds to two Smaug Response Elements (SREs) in the *nos* 3’ UTR (58–61). Smaug also induces *nos* mRNA degradation during the first 2 $\frac{1}{2}$ h of embryo development (59,62).

Smaug and the related yeast protein Vts1p effect deadenylation of their RNA targets by the CCR4–NOT complex (21,63–65). Smaug-dependent deadenylation contributes to the large-scale destruction of maternal mRNAs as part of the maternal-to-zygotic transition, which transfers the control over the embryo from the maternal to the zygotic genome (66). Physical and genetic interactions between Smaug and the CCR4–NOT complex have been observed repeatedly (64,67–69), but since the interaction has not been examined with purified proteins, it is unclear whether it is direct. Among the many mRNAs deadenylated under the influence of Smaug is *nos*, which has a short poly(A) tail at steady-state (21,67,70–72).

Stimulation of CCR4–NOT-catalyzed deadenylation by specificity factors has so far only been reconstituted with *S. pombe* CCR4–NOT and three different effectors (37,44). The data were consistent with a simple ‘tethering’ model in which an individual effector protein associates with a specific RNA sequence and recruits CCR4–NOT by direct interaction. The question how Smaug induces mRNA deadenylation is more complicated, as Smaug does not act on SRE-containing RNAs by itself. Rather, it initiates the assembly of a repressor complex containing six other proteins (68,73), all of which are conserved: Cup is an oocyte- and embryo-specific paralog of 4E-T and repressor of translation (74–77). In tethering experiments in cultured cells, Cup induces deadenylation of the bound mRNA and co-precipitates with the CCR4–NOT complex (69,78) but a direct interaction has not been demonstrated. Whether 4E-T also induces deadenylation has been controversial (79,80). A third constituent of the Smaug-dependent repressor complex is Me31B (DDX6 in mammals) (68,73,81). Me31B associates with the MIF4G domain of NOT1 on a surface opposite the CAF1 binding site (33,34,82), but Me31B-dependent deadenylation has, to our knowledge, not been reported. Me31B also binds Cup, suggesting the possibility that Cup-dependent deadenylation might be mediated by Me31B (83–86). Four additional proteins are part of the Smaug-dependent repressor complex (68,73): Trailer hitch (Tral; Lsm14 in mammals), one of several proteins associating with Me31B in a mutually exclusive manner (87–89); the translation initiation factor eIF4E, which is bound by Cup (75–77,90,91); the cytoplasmic poly(A) binding protein, PABPC; and the DEAD-box protein Belle (DDX3 in mammals). Among these proteins, PABPC can facilitate deadenylation by CCR4–NOT; specifically, PABPC has been reported to promote the activity of CCR4 but inhibit CAF1 (26,92). However, since PABPC is thought to be bound to poly(A) tails in general, it is unlikely to contribute specifically to SRE-dependent deadenylation. Deadenylation of *nos* is disturbed in *belle* mutants (68), but a direct involvement of Belle in deadenylation has not been examined. Tral and eIF4E have not been tested for a role in deadenylation.

In this paper we have used biochemical reconstitution assays to address the question whether Smaug can accelerate deadenylation as an individual protein, by direct recruitment of the CCR4–NOT complex, and/or whether additional components of the Smaug-dependent translation repressor complex are also employed for the purpose of deadenylation. We find that both Smaug and Cup, individually and cooperatively, directly promote deadenylation by the CCR4–NOT complex.

## MATERIALS AND METHODS

### Expression clones and viruses

Co-expression of two or more proteins from one baculovirus vector was performed by means of the MultiBac expression system (93,94). For expression of the ^Dm^CCR4–NOT variants, all ORFs were amplified from *D. melanogaster* cDNA. For NOT1_MINI_ (amino acid residues 1147-2505), NOT2, NOT3 and CAF1, N-terminal His_8_-tags were added by PCR. C-terminal FLAG-tags were appended to CCR4 and CAF40 by PCR. His_8_-NOT2 was cloned into the pFBDM vector between the SmaI and KpnI restriction sites. His_8_-NOT1_MINI_ was first introduced between the NheI and HindIII restriction sites of the pET28a-MBP plasmid, then cut out with PspOMI and HindIII and inserted into the pFBDM-His_8_-NOT2 plasmid between the NotI and HindIII restriction sites. This pFBDM-His_8-_NOT2_His_8-_NOT1_MINI_ plasmid was the basis for the generation of longer NOT1 variants lacking the His-tag: Additional parts of NOT1 were inserted into the NOT1_MINI_ plasmid between the XbaI and AgeI restriction sites until the full length NOT1 was obtained: AvrII_NOT1_MINI_EXT__AgeI (residues 878–1570); XbaI_NOT1_pE__AgeI (residues 288–1570); XbaI_NOT1_pC__AgeI (residues 1–1570). CAF40-FLAG was cloned into the pFBDM vector between the EcoRI and HindIII restriction sites. His_8_-NOT3 was then inserted between the SmaI and KpnI restriction sites of pFBDM-CAF40-FLAG, resulting in the pFBDM-CAF40-FLAG_His_8_-NOT3 plasmid. For the nuclease module, CCR4-FLAG was inserted in the pFBDM vector between the XhoI and KpnI restriction sites. His_8_-CAF1 was then cloned between the BamHI and XbaI restriction sites of pFBDM-CCR4-FLAG, resulting in the pFBDM-CCR4-FLAG_His_8_-CAF1 plasmid. Point mutations in CCR4 (412D/414N to alanine) and CAF1 (53D/55E to alanine) (69) were introduced by overlap extension PCR. NOT10 and NOT11 cDNAs, cloned in pnYC-NpM and pnEA-CvH, respectively (28) were obtained from Eugene Valkov. The NOT10 sequence was removed from its vector with KpnI and NheI and inserted between the KpnI and AvrII sites of pnEK-His_8_-MBP-Cup (described below), replacing the Cup ORF. Thereby, an N-terminal His_8_-MBP-tag followed by a HRV 3C protease site was fused to NOT10. A BglII-XbaI fragment containing His_8_-MBP-NOT10 was then cut out of the pnEK vector and cloned between the BglII and AvrII restriction sites behind the second T7 promoter of the pETDuet-1 vector (Merck, Darmstadt, Germany). The resulting plasmid was cut with SalI and BglII, and NOT11, cut as an XhoI-BamHI fragment from its vector, was introduced behind the first T7 promoter. This created a fusion with an N-terminal His_6_-tag in the pETDuet-1-His_8_-MBP-NOT10_His_6_-NOT11 plasmid.

DNA sequences coding for the putative constituents of the SRE-dependent repressor complex were also amplified from *D. melanogaster* cDNA, except Cup, which was a kind gift of Fulvia Bono (in the pnEK vector (95)). C-terminal FLAG-tags were added to Smaug and Cup by PCR. Smaug-FLAG was inserted between the NheI and NotI restriction sites of the pFBDM vector. Cup-FLAG was cloned between the SmaI and XhoI restriction sites of the pFBDM vector. For the generation of MBP-tagged Cup fragments, MBP was cut out of pnYC-NpM with SpeI and XhoI and inserted between the equivalent sites of pnEK-Cup. An N-terminal His_8_ tag was cloned, as a synthetic oligonucleotide, into the NcoI site of pnEK-MBP-Cup. The resulting plasmid, pnEK-His_8_-MBP-Cup, was the basis for the generation of truncated Cup variants (N: aa 1–417; M: aa 418–770; C: aa 771–1117; and combinations thereof), which were amplified from pFBDM-Cup-FLAG and inserted between the XhoI and AvrII restriction sites of pnEK-His_8_-MBP-Cup for *E. coli* expression. MBP-Cup fragments were cut out of the pnEK-His_8_-MBP vector with NcoI and AvrII and inserted into the pFBDM vector between the NcoI and NheI restriction sites. An N-terminal His_8_-λN-tag was introduced as a restriction fragment into the pFBDM-MBP-Cup fragments using the NcoI and SmaI sites, resulting in the pFBDM-His_8_-λN-MBP-Cup fragment plasmids. The GST-Me31B clone in pFastBac1 (Thermo Fisher) has been described (68). A C-terminal His_8_-tag was appended to Tral by PCR, and the resulting fragment was cloned into pFastBac1 with EcoRI and NcoI. For expression of His-T7-Tral-His in *E. coli*, the coding sequence was cut out from the pFastBac 1 construct and transferred to pET28a (Merck, Darmstadt, Germany) by means of HindIII and Eco53kI. An N-terminal His_8_-tag was added to Belle by PCR, and His_8_-Belle was inserted between the XhoI and HindIII restriction sites of the pFastBac1 vector. PABPC was cloned into pET-28a using BamHI and XhoI.

All expression constructs were confirmed by Sanger sequencing. Accession numbers for the sequences amplified are listed in Supplementary Table 1, and all primers used to generate expression constructs are listed in Supplementary Table 2.

pFastBac1 and pFBDM DNA constructs were transformed into DH10MultiBac cells (Geneva Biotech) and incubated for 5 h to allow transposition into the mini-*att*Tn7 sites of the bacmid DNA. Colonies were selected for correct bacmid DNA by antibiotic resistance and blue-white screening. Bacmid DNA was isolated via alkaline lysis (Qiagen buffers P1, P2 and P3) and isopropanol precipitation. Sf21 cells (1.5 × 10^6^ cells seeded into a 6-well plate) were transfected with 10 μg bacmid DNA, which had been pre-incubated in 200 μl Ex-Cell 420 medium plus 5 μl FuGENE HD transfection reagent (Promega) for 20 min at 27°C. After 96 h, the supernatant was collected and used to infect 0.8 × 10^6^ Sf21 cells/ml at a 1:500 volume ratio for virus propagation (V1 generation). The V1 generation was used to infect Sf21 cells for protein expression.

### Protein expression and purification

Anti-FLAG M2 agarose and FLAG peptide were from Sigma, Ni-NTA agarose was from Qiagen, amylose resin from NEB, and Glutathione Sepharose 4B and the Superose 6 column from Cytiva. PES concentrators were from Thermo Fisher and Amicon concentrators from Merck, Darmstadt, Germany. HRV 3C protease was purified in-house by Bodo Moritz. Sf21 cells were grown as suspension cultures in Ex-Cell 420 serum-free medium (Sigma-Aldrich) at 27°C.

For the reconstitution of hexameric ^Dm^CCR4–NOT complexes, Sf21 cells were co-infected with a baculovirus expressing His_8_-NOT3 + CAF40-FLAG, a second virus expressing CCR4-FLAG + His_8_-CAF1, and a third virus expressing His_8_-NOT2 with one of the different NOT1 variants. The baculoviruses were used at a 2:1:2 ratio. Cells were harvested 72 h after infection, resuspended in ice-cold lysis buffer (500 mM NaCl, 50 mM HEPES–NaOH and 10 mM potassium phosphate, pH 7.6, 10% sucrose, 1 mM PMSF, 1 μM pepstatin A). Sucrose stabilized the CCR4–NOT complex during freezing and thawing. Cells were lysed by sonication on ice, the lysate was cleared by centrifugation for 20 min at 20.000 x g and the supernatant applied to anti-FLAG M2 agarose matrix for 2 h under constant rotation at 6–8°C. The matrix was washed four times with wash buffer (lysis buffer minus PMSF and pepstatin A) in a batch format, then protein was eluted by addition of wash buffer with 200 μg/ml FLAG peptide. The eluate was collected after 30 min, concentrated with a PES concentrator (10 kDa MWCO) and applied to a Superose 6 column equilibrated in wash buffer. Removal of the filter from the Superose column improved protein recovery. Fractions containing the hexameric complex were pooled, concentrated as before and frozen in liquid nitrogen before final storage at –80°C.

For reconstitution of the octameric ^Dm^CCR4–NOT_FULL_ complex, His_8_-MBP-NOT10 and His_6_-NOT11 were co-expressed in *E. coli* BL21 Star (DE3) cells with 24 h lactose autoinduction (96). Cells were harvested, resuspended in ice-cold lysis buffer containing 1 mg/ml lysozyme and 10 mM imidazole and incubated for 1 hour under constant rotation at 6–8°C. Cells were then sonicated, the lysate was centrifuged for 20 min at 15 000 × g and the supernatant applied to Ni-NTA agarose for 2 h under constant rotation at 6–8°C. The matrix was washed four times with wash buffer plus 20 mM imidazole, and proteins were eluted with wash buffer plus 160 mM imidazole. After 30 min, the eluate was applied to amylose resin equilibrated in wash buffer and incubated for 2 h. NOT10 and His_6_-NOT11 were eluted by a 2 h incubation with an approximately equimolar amount of HRV 3C protease and concentrated with a PES concentrator (10 kDa MWCO). A twofold excess of the heterodimer was incubated for 2 h at 8°C with the hexameric CCR4–NOT (isoform NOT1_PC_) to reconstitute ^Dm^CCR4–NOT_FULL_, which was purified on a Superose 6 column, concentrated and stored like the hexameric complex.

For production of Smaug-FLAG, Cup-FLAG, Tral-His_8_, GST-Me31B or His_8_-Belle, Sf21 cells were infected with the relevant baculoviruses. Infected cells were processed as above up to the addition of the affinity matrix. Binding to the respective matrix (anti-FLAG M2 agarose, Ni-NTA agarose, or Glutathione Sepharose 4B) was carried out for 2 h, then the matrix was washed four times with wash buffer (with 20 mM imidazole for His_8_-tagged constructs), and proteins were eluted by wash buffer containing 200 μg/ml FLAG peptide or 160 mM imidazole or by on-column cleavage with HRV 3C protease for Me31B. After elution, the proteins were, if necessary, concentrated with an Amicon Ultra concentrator (10 kDa MWCO), flash frozen and stored at –80°C. MBP- and His_8_-λN-MBP-Cup fragments were also expressed in Sf21 cells by baculoviral infection. Infected cells were processed as above up to the addition of the affinity matrix. His-MBP-tagged proteins were first purified by Ni-NTA affinity chromatography as above and then applied for 2 h to an amylose agarose matrix. Bound proteins were eluted with wash buffer containing 20 mM maltose. MBP-Cup fragments were purified by direct addition of cell lysates to the amylose matrix.

*E. coli* BL21 Star (DE3) cells were used for the expression of His_6_-T7-PABPC and His_6_-T7-Tral-His_8_. Expression and Ni-NTA chromatography were carried out as described for the NOT10–NOT11 purification, except that the buffer contained no phosphate. At this point, Tral and PABPC were flash frozen and stored at –80°C.

Identities of purified proteins were confirmed by western blotting. Protein concentrations were determined by SDS-polyacrylamide gel electrophoresis and Coomassie staining in comparison to a set of BSA standards. Intensities of protein bands were evaluated with Fiji. For deadenylation experiments, proteins were pre-diluted in 200 mM potassium acetate, 50 mM HEPES–KOH, pH 7.4.

### Western blotting

Proteins were transferred onto nitrocellulose membrane (GE Healthcare) by wet blotting overnight at 8°C and 27 V. Membranes were blocked with 1.5% gelatin (from cold water fish skin; Sigma-Aldrich) in 1x TBS - 0.05 % Tween and incubated with the primary antibody for 2 h. The antibody against Smaug (97) was diluted 1:500 in blocking buffer. The membrane was washed three times with 1× TBS–Tween and incubated with fluorescently labelled secondary antibody (IR-Dye, LI-COR; 1:15000). The membrane was washed again three times with 1× TBS–Tween and scanned with a LI-COR Odyssey imager.

### Deadenylation substrates

The SRE-only RNA and the TCE RNA as well as their mutant (SRE^MUT^) versions have been described (72). The TCE RNA was called *nos* RNA in (72). The plasmid vector for these RNAs also encoded a poly(A) tail of about 70 nt. Compared to the version described (72), it was modified by introduction of a BsaI site into the HindIII site at the end of the poly(A) tail, so that, upon BsaI digestion, a run-off transcript was produced that ended in a straight poly(A) tail without additional nucleotides from the restriction site. The BoxB tethering construct was generated by assembly of a synthetic BamHI-XbaI restriction fragment containing two BoxB elements (98) (Supplementary Table 2) and insertion into the BglII–XbaI restriction sites of the pBSK-nLuc-nos plasmid (99). The control construct (nLuc-BRE^MUT^-A_70_ RNA) contained the mutated AB Bruno recognition element (BRE) of the *oskar* 3’ UTR (100). The pBSK-nLuc-BRE^WT^ plasmid was first generated by amplifying the BRE from *Drosophila* cDNA and introducing it between the BglII-XbaI restriction sites of the pBSK-nLuc-nos plasmid. The mutant BRE was then assembled from four synthetic DNA oligonucleotides (Supplementary Table 2) and introduced downstream of the nLuc ORF between the BamHI and EcoRI sites of the pBSK-nLuc-BRE^WT^ plasmid, replacing the WT BRE.

RNAs were synthesized with T3 RNA polymerase (Promega) in the presence of [α-^32^P]UTP and 7 mM ‘anti-reverse’ cap analog (m^7,3'-O^GpppG; Jena Bioscience). All radiolabeled RNAs were gel-purified. Alternatively, the RNA was labeled by incorporation of a 6-carboxyfluorescein- (6-FAM-) labelled ApG cap analog. The cap analog was added to the transcription reaction at 1 mM in the absence of GTP. After a 5 min preincubation, GTP was added to 0.2 mM, and the incubation was continued for an additional 20 min before the GTP concentration was raised to 1 mM. After an additional hour, the reaction was incubated with 1 U DNase I (Roche), the RNA was phenol-extracted and ethanol precipitated and used without further purification.

The chemically synthesized FAM 7mer-A_20_ RNA has been described (27).

### Deadenylation assays

Reactions were carried out in 20 μl or multiples thereof for time-courses. The reaction buffer consisted of 50 mM potassium acetate, 30 mM HEPES–KOH, pH 7.4, 2 mM magnesium acetate, 0.15 mg/ml nuclease-free BSA (Merck-Millipore), 2 mM DTT, 800 U/ml murine RNase Inhibitor (NEB) and 3% (w/v) PEG 20000. The presence of BSA in the buffer improved the stability of the CCR4–NOT complex during extended incubations at low concentration. Yeast tRNA (0.25 mg/ml) was added as a carrier. Reactions with the FAM-7mer-A_20_ RNA did not contain tRNA, unless noted otherwise. Substrate RNA was either directly incubated with CCR4–NOT complexes or pre-incubated for 20–30 min with potential activators of deadenylation at 25°C, and deadenylation was started by addition of CCR4–NOT. Reaction temperature was 25°C both for the human and the *Drosophila* CCR4–NOT complex. The reaction was stopped by addition of a two- to threefold excess of ice-cold formamide loading buffer (95% deionized formamide, 17.5 mM EDTA, pH 8.0, 0.01% bromophenol blue, 0.01% xylene cyanol). Xylene cyanol was omitted for FAM-7mer-A_20_ substrates. Samples were heated to 95°C for 3 min, cooled on ice and separated on denaturing polyacrylamide gels (19:1 acrylamide-bis acrylamide, 1× TBE, 7 M urea). Fluorescent reaction products were directly visualized by scanning with a Typhoon 9200, whereas gels with radioactive RNA were placed on storage phosphor screens overnight at –20°C and the screens scanned the next day. Images were analysed using Fiji (101). Deadenylation rates were determined as described (102). Briefly, the modal poly(A) tail length at each time point was determined by densitometric analysis of the deadenylation pattern in each lane using Fiji. Then the modal poly(A) tail length was plotted over time and the deadenylation rate (in nucleotides/min) was derived from the slope of the fit line.

The number of experiments (*n*) reported in the figure legends refers to independently performed assays. In most cases, this included the use of at least two independent protein preparations.

### RNA binding assays

His_8_-MBP- or MBP-tagged Cup or Cup fragments (0.6 pmol) were incubated with a 5’-^32^P-labeled RNA oligonucleotide (GGGTTTAGTGCGCACGTG, 18nt; 0.5 pmol) in 10 μl 16 mM HEPES, pH 7.6, 50 mM potassium acetate, 1 mM magnesium acetate, 0.8 mM ATP, 0.24 mg/ml yeast tRNA, 1 mM DTT, 10% glycerol for 15 min at room temperature and UV crosslinked (Stratalinker 1800, 254 nm, 200 mJ/cm^2^) on ice. Reaction products were separated on SDS-polyacrylamide gels, which were dried and analyzed by phosphoimaging. For analysis of the cross-link product by affinity purification, a 10 x binding reaction was set up, and a part of each reaction was stored as input. The remaining portions were diluted in 50 mM HEPES, pH 7.6, 500 mM KCl, 10% sucrose, 6 M urea, 0.05% NP40 and incubated for 15 min at room temperature. Magnetic Ni-NTA beads (Promega) were added to the reaction, and the mix was incubated at room temperature for 1 h on a rotating wheel. The matrix was collected and washed twice with buffer as above. Before elution in SDS sample buffer, the matrix was transferred to a fresh tube. Input and elution fractions were analyzed by gel electrophoresis as above. For technical reasons, positions of protein standards indicated next to the gels are approximate.

For electrophoretic mobility shift assays, binding reactions were carried out in deadenylation reaction buffer, including tRNA, plus 5% glycerol for 20–30 min at 25°C. RNA-protein complexes were separated on nondenaturing polyacrylamide gels (5% 60:1 acrylamide-bis acrylamide, 0.5x TBE) and visualized by phosphoimaging.

### Protein-protein interaction assays

ReLo assays were performed as described (103). In short, *Drosophila* S2R+ cells were seeded onto four-well chambered coverslips (Ibidi), co-transfected with the desired combination of pAc5.1 plasmids expressing the two proteins of interest, and protein localization was analyzed after two days by live confocal fluorescence microscopy. Split-ubiquitin yeast two-hybrid experiments were performed as described (104). The desired combinations of bait and prey plasmids were co-transformed into NMY51 cells and plated onto SC agar lacking Leu and Trp. For the spotting assay, several colonies were pooled to prepare an overnight liquid culture. Three 10-fold dilutions were spotted onto SC agar plates either lacking Trp and Leu (control) or Trp, Leu, His and adenine (selection), and, after three days of growth, images were taken. Detailed information on all plasmids used for protein-protein interaction assays is provided in Supplementary Table 3.

To test for an interaction between Smaug and the CCR4–NOT complex by gel filtration, Smaug (15 μg in 50 μl) was incubated either with ^Dm^CCR4–NOT_MINI_ (15 μg in 20 μl) or protein buffer for 1 h at 8°C. 50 μl of the mixture was applied to a Superose 6 gel filtration column (bed volume ∼ 2.4 mL; Cytiva) equilibrated in lysis buffer lacking PMSF and pepstatin A. The column was run with the same buffer at 0.017 column volumes per min. Column fractions were analyzed by SDS polyacrylamide gel electrophoresis and Coomassie staining and/or by western blotting with an antibody against Smaug.

## RESULTS

### Reconstitution of the CCR4–NOT complex from *Drosophila melanogaster*

In order to study the mechanism of Smaug-induced deadenylation, we reconstituted and purified five different versions of the *Drosophila* CCR4–NOT complex (Figure 1A). Experiments were guided by earlier work on the human complex (27). *^Dm^*CCR4–NOT_FULL_ was composed of full-length versions of all eight subunits (CAF1, CCR4, NOT1-3, CAF40, NOT10 and NOT11).*^Dm^*CCR4–NOT_Δ10:11_ lacked NOT10 and 11. *^Dm^*CCR4–NOT_PE_ was similar, but contained the naturally occurring NOT1 splice variant PE, which lacks the 5’ portion of the open reading frame (https://flybase.org). In *^Dm^*CCR4–NOT_MINI_EXT_, NOT1 was further shortened and started at amino acid 877. *^Dm^*CCR4–NOT_MINI_ contained the shortest NOT1 version, starting at amino acid 1147 and thus missing the tristetraprolin binding site mapped in the mammalian ortholog (43). For the production of these complexes, three MultiBac clones were generated, containing NOT1 and NOT2; NOT3 and CAF40-FLAG; or FLAG-CCR4 and CAF1, respectively (93,94). Complexes composed of all six subunits were produced by co-infection of insect cells with the three viruses and purified by Flag affinity-purification followed by gel filtration. For the production of ^Dm^CCR4–NOT_FULL_, His_6_-MBP-NOT10 and His_6_-NOT11 were co-expressed in *E. coli*. After co-purification, they were mixed with the Flag-purified six subunit complex, and the assembly was purified by gel filtration. The resulting preparations were pure and generally contained approximately stoichiometric amounts of the subunits (Figure 1B; C, left panel). Excess CCR4-CAF1 heterodimer was obtained from the same gel filtration columns (Figure 1B; C, left panel). The basal deadenylation activities of these complexes were measured in reactions with a small synthetic RNA substrate (‘FAM 7mer-A_20_’: seven nt ‘body’ plus 20 3’-terminal A residues) (27), carrying a fluorescent label at its 5’ end. Activities of the five larger complexes were similar (Figure 1D). We conclude that NOT10, NOT 11, and the N-terminal half of NOT1 do not increase the basal activity of CCR4–NOT. In contrast, CAF40 and/or the NOT module promote deadenylation, as the activity of the CCR4-CAF1 heterodimer was about 2% of that of the MINI complex (250 nM of the heterodimer had an activity similar to 5 nM of the MINI complex; Supplementary Figure 1A). This is in qualitative agreement with reports for the *S. pombe* and human CCR4–NOT complexes (27,37,38). The activity of CCR4–NOT_MINI_ was highest at ∼1 mM Mg^2+^ and ∼50 mM potassium acetate (Supplementary Figure 1B).

### Smaug is sufficient to induce SRE-dependent deadenylation by the CCR4–NOT complex

In order to examine Smaug-dependent deadenylation in a fully reconstituted system, we overproduced and purified Smaug and its associates: Smaug, Cup, Me31B and Belle were produced in insect cells by means of baculovirus vectors, PABPC was made in *E. coli*, and Tral was made in either system (Figure 1C, right panel). Cup was the least pure among all proteins. Attempts at further purification were not successful as the protein, after elution from the initial Flag affinity column, did not elute in a defined peak from any other column tested.

Short substrate RNAs were used to assay for Smaug-dependent deadenylation: Most experiments employed the ‘SRE-only’ RNA, which contained two synthetic SREs, either wild-type (SRE^WT^) or with a single inactivating point mutation in each (SRE^MUT^) (72). Both RNAs carried a plasmid-encoded poly(A) tail of some 70 nucleotides. Linearisation of the plasmid DNA for run-off transcription was such that no non-A nucleotides were encoded at the end of the poly(A) tail (see Materials and Methods).

**Figure 2.**
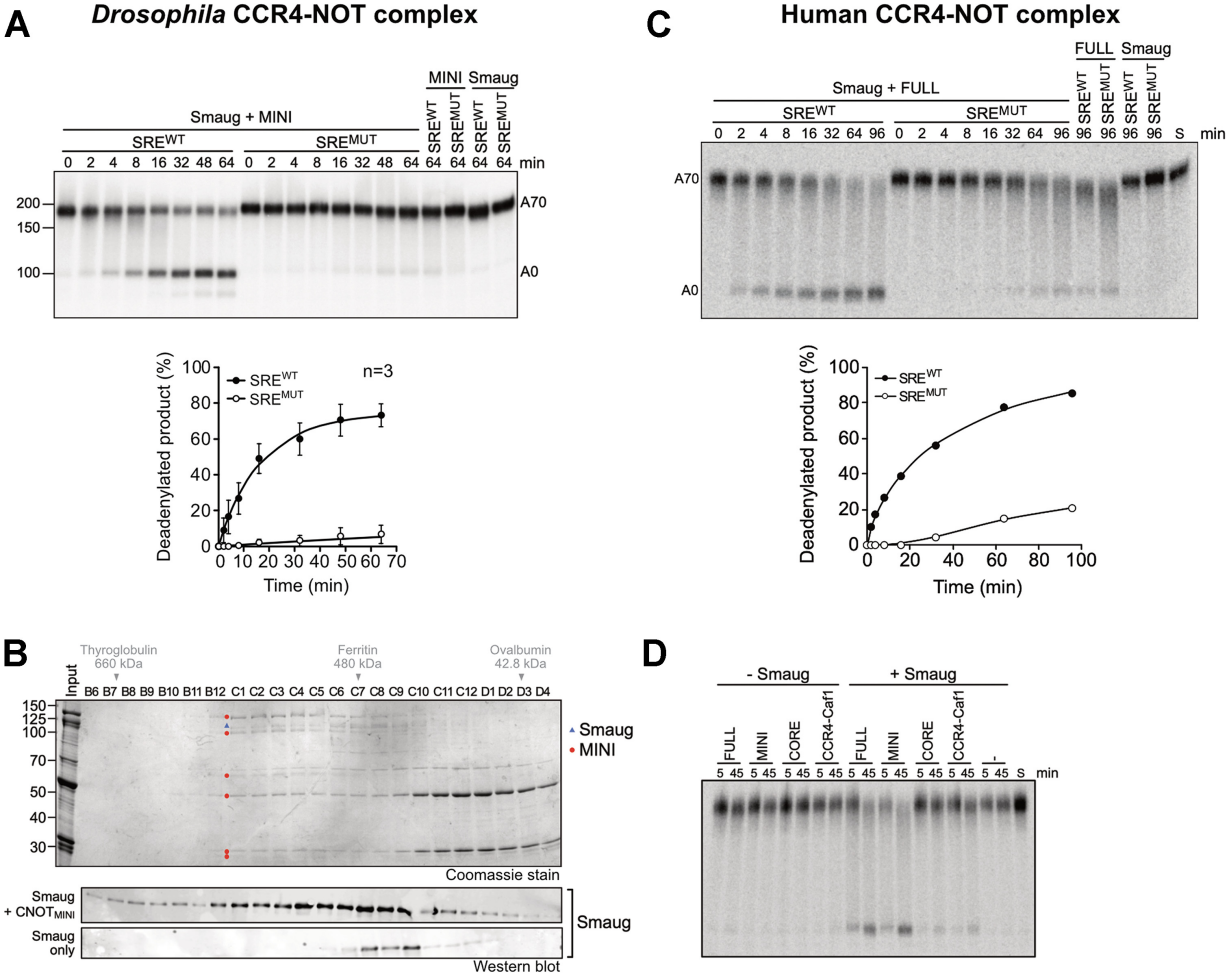
Smaug is sufficient to induce SRE-dependent deadenylation by the CCR4–NOT complex. (**A**) Smaug induces deadenylation by the ^Dm^CCR4–NOT_MINI_ complex. Radioactively labelled SRE^WT^-A_70_ or SRE^MUT^-A_70_ RNAs (20 nM) were pre-incubated with 80 nM Smaug or deadenylation buffer. Reactions were started by the addition of 2 nM ^Dm^CCR4–NOT_MINI_, and samples were taken and analyzed at the times indicated. Reactions containing only CCR4–NOT or only Smaug were included as controls. The graph at the bottom represents the time-dependent accumulation of fully deadenylated RNA (average plus/minus standard deviation based on *n* = 3). (**B**) Gel filtration reveals an association of Smaug with the ^Dm^CCR4–NOT_MINI_ complex. Smaug by itself or mixed with ^Dm^CCR4–NOT_MINI_ was analyzed by gel filtration as described in Material and Methods. Column fractions from the samples containing both CCR4–NOT and Smaug were analyzed by SDS polyacrylamide gel electrophoresis and Coomassie staining (top panel) and by western blotting with an antibody against Smaug (middle panel). Column fractions derived from the Smaug-only sample were only analyzed by western blotting (bottom panel). Elution positions of size markers are indicated above the respective fractions. In the Coomassie-stained gel, Smaug is labeled with a blue triangle, and subunits of the CCR4–NOT complex are labeled with red dots. (**C**) Smaug induces SRE-dependent deadenylation by the human CCR4–NOT complex. Reactions were carried out as in Figure 2A except that RNA was used at 5 nM, Smaug at 30 nM, and the human CCR4–NOT_FULL_ complex at 10 nM. The graph at the bottom represents the time-dependent accumulation of fully deadenylated RNA. A representative experiment of *n* = 2 is shown. The requirement for a higher concentration of CCR4–NOT compared to the experiment in Figure 2A is at least partially explained by the reaction temperature of 25°C, which is suboptimal for the human complex. These reactions were also carried out in the absence of BSA. (**D**) The CCR4–NOT ‘MINI’ complex is necessary and sufficient for Smaug-dependent deadenylation. 5 nM ^32^P-SRE^WT^-A_70_ RNA was preincubated with 30 nM Smaug or buffer, then deadenylation was initiated by the addition of the different human CCR4–NOT complexes (10 nM of Full, Mini and Core; 50 nM of CCR4-Caf1). Samples were taken and analyzed at the times indicated. A representative experiment of *n* = 2 is shown.

SRE-only RNAs were mixed with Smaug and preincubated to allow complex formation. Addition of ^Dm^CCR4–NOT_MINI_ resulted in rapid deadenylation of the SRE^WT^ RNA during an incubation at 25°C; in the SRE^MUT^ control, the fully deadenylated product appeared only after a lag phase of ∼16 min and then accumulated at a ∼ 50fold lower rate compared to SRE^WT^ (Figure 2A). The ^Dm^CCR4–NOT_FULL_ complex behaved similar to MINI (Supplementary Figure 1C). Three observations indicate that the RNA shortening visible in the gel was due to deadenylation: (1) The reaction product had the size expected for deadenylation. (2) Results similar to those seen with internally labeled RNA in Figure 2A were also obtained with 5’-end-labeled RNA (see below, Figure 6B). (3) The reaction was affected by point mutations in the active sites of the deadenylases CCR4 and CAF1 (see below, Figure 4). As negative controls, Smaug by itself did not catalyze deadenylation, and the CCR4–NOT complex by itself had a barely detectable basal deadenylation activity (Figure 2A). As a further negative control, human PTB, an RNA binding protein and regulator of splicing (105), did not accelerate deadenylation (data not shown). Several constituents of the SRE-dependent repressor complex were also inactive (see below, Supplementary Figure 6). Thus, stimulation of CCR4–NOT-dependent deadenylation by Smaug is a specific effect. Smaug-dependent deadenylation in embryo extract has previously been found to be sensitive to ATP depletion (72). However, the reconstituted deadenylation reaction proceeded in the absence of ATP and was not affected by its addition (data not shown). Smaug-dependent deadenylation was stimulated by the presence of polyethylene glycol (PEG 20000), presumably due to a macromolecular crowding effect (Supplementary Figure S2A). PEG was therefore routinely included in the deadenylation buffer. The activity of the CCR4–NOT complex itself was not affected by PEG (data not shown). miRISC-dependent deadenylation has been reported to be associated with liquid-liquid phase separation (LLPS) (106), and LLPS is promoted by crowding reagents like PEG (107). In miRISC-dependent deadenylation, LLPS causes rapid sedimentation of protein–RNA complexes in a microcentrifuge (106). By this criterium, no LLPS was detectable in our deadenylation assays.

### Smaug interacts with NOT3

The data so far demonstrate that Smaug induces SRE- and CCR4–NOT-dependent deadenylation independently of its associated repressor proteins. This would be most easily explained by SRE-bound Smaug recruiting the deadenylase through a direct interaction. Such an interaction was in fact demonstrated by analytical gel filtration (Figure 2B): Smaug alone was recovered in low yields; only small amounts were detectable by western blotting. These eluted in fractions C8–C10, somewhat ahead of the position expected based on the molecular weight of monomeric Smaug. When Smaug was analyzed together with ^Dm^CCR4–NOT_MINI_, recovery was improved, Smaug was detectable by Coomassie staining, and a large fraction of the protein co-eluted with CCR4–NOT in fractions C1–C6.

To define components of CCR4–NOT required for the interaction with Smaug, we made use of different variants of the human deadenylase complex (27). Smaug induced SRE-dependent deadenylation by the ^Hs^CCR4–NOT_FULL_ complex (Figure 2C). This eight subunit complex is essentially complete except for an N-terminal deletion of NOT11 (27). ^Hs^CCR4–NOT_MINI_, corresponding to ^Dm^CCR4–NOT_MINI_ except for N-terminal deletions of NOT2 and NOT3, catalyzed Smaug- and SRE-dependent deadenylation with an efficiency comparable to the FULL complex (Figure 2D). CCR4–NOT_CORE_ is a complex further simplified by omission of the NOT module (NOT2, NOT3 and a C-terminal part of NOT1) and thus consists only of CAF1, CCR4 and CAF40 bound to a central NOT1 fragment. CCR4–NOT_CORE_ responded to Smaug very weakly. A CCR4–CAF1 heterodimer behaved similarly (Figure 2D). We conclude that the Smaug interaction surface of the CCR4–NOT complex is conserved between *D. melanogaster* and *H. sapiens* and is largely if not entirely contained in the NOT module.

**Figure 3.**
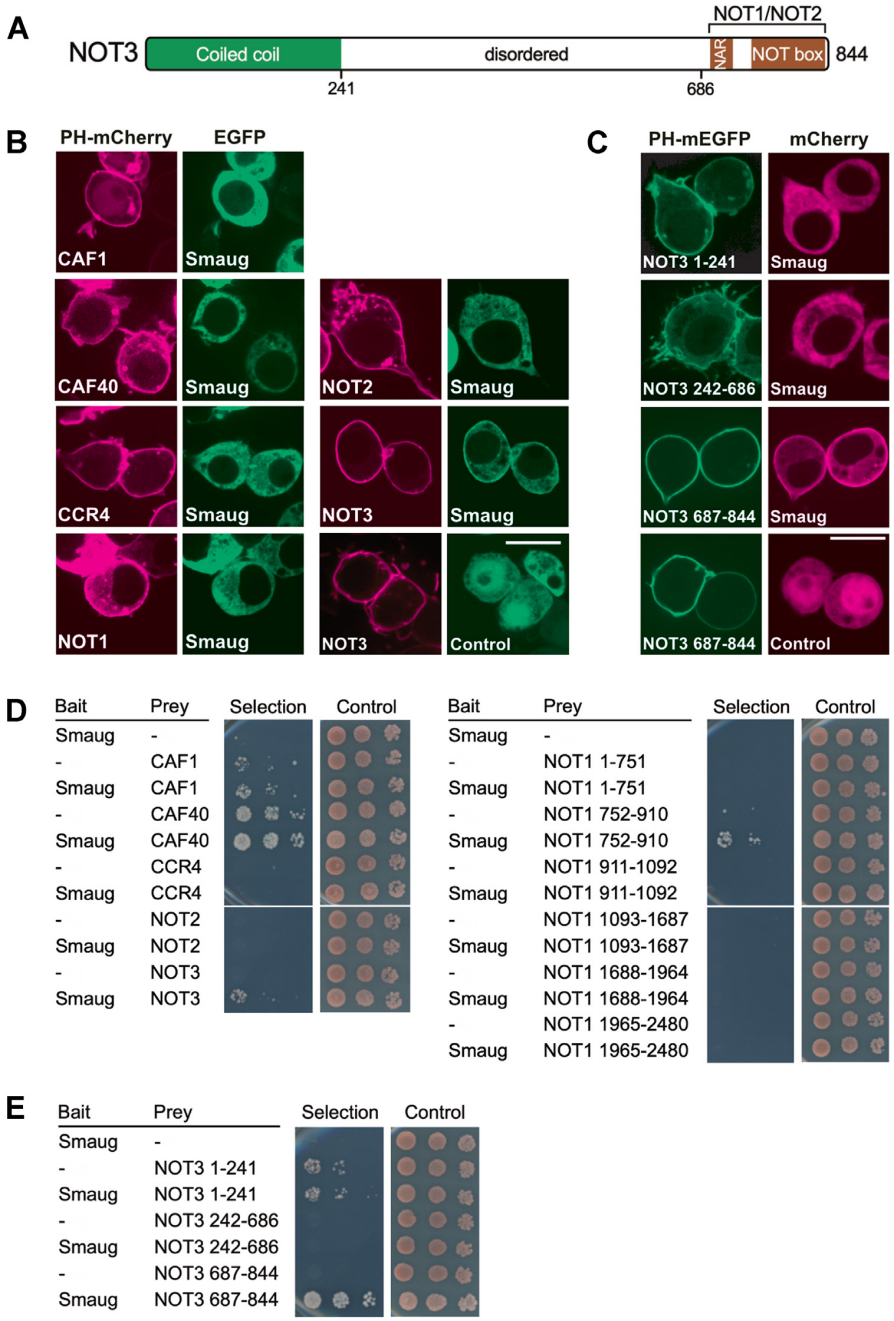
Smaug associates with CCR4–NOT via NOT3. (**A**) Domain structure of NOT3. NAR, NOT1 anchor region. The NOT box mediates the interaction with NOT2. Borders of fragments used in the interaction assays are indicated at the bottom. (**B**) Smaug interacts with NOT3 in a relocalization assay. *Drosophila* proteins were fused with PH-mEGFP or mCherry as indicated and transiently coexpressed in *Drosophila* S2R+ cells. After two days, subcellular protein localization was examined by confocal live fluorescence microscopy. Smaug relocalization was detected only with the NOT3 subunit. ‘Control’ indicates a plasmid expressing EGFP only. Scale bar is 10 μm. Additional images supporting the Smaug – NOT3 interaction are shown in Supplementary Figure 3A. (**C**) The Smaug interacting surface is in the C-terminal domain of NOT3 as determined with the ReLo assay. NOT3 fragments were used as bait fusions as indicated. Fluorescent tags were swapped in comparison to (**B**). ‘Control’ indicates a plasmid expressing mCherry only. Scale bar is 10 μm. Additional images supporting the interaction of Smaug with the C-terminal fragment of NOT3 are shown in Supplementary Figure 3B. (**D**) Smaug interacts with NOT3 in a yeast two-hybrid assay. Split-ubiquitin yeast two-hybrid assays were performed with bait and prey constructs containing *Drosophila* proteins as indicated or no insertion (–). Three 10-fold dilutions of the cells were spotted and imaged after three days of incubation. Selection medium lacked histidine and adenine. (**E**) Yeast two hybrid assay confirms interaction of Smaug with the C-terminal domain of NOT3. The same fragments as in (**C**) were used as prey fusions in the two-hybrid assay.

Interactions between Smaug and individual CCR4–NOT subunits present in the MINI complex were then examined by a recently described *in vivo* interaction assay based on intracellular protein relocalization (ReLo assay) (103). For this assay, CCR4–NOT subunits were fused with mCherry and with the pleckstrin homology (PH) domain of rat phospholipase Cδ1 and expressed as bait in *Drosophila* S2R+ cells; the PH domain caused their localization on the plasma membrane. Smaug, as potential prey protein, was labeled with EGFP. Whereas EGFP-Smaug by itself is expected to be cytoplasmic, an interaction with one of the bait proteins is expected to become visible as the co-localization of the two fluorescence markers on the plasma membrane. With five out of the six bait proteins tested, Smaug remained widely distributed in the cytoplasm. Membrane-bound NOT3, however, caused a clear re-localization of Smaug to the plasma membrane, indicating an interaction (Figure 3B, Supplementary Figure 3A). The Smaug interaction surface of NOT3 was mapped by additional ReLo experiments with swapped tags: NOT3 fragments (Figure 3A), fused to the PH domain and mEGFP, were combined with mCherry-tagged Smaug. The results indicated that Smaug binding is mediated by the C-terminal region of NOT3 (amino acids 687–844), which contains the NOT1-interacting region and the NOT box (Figure 3A, C, Supplementary Figure 3).

A split-ubiquitin yeast two-hybrid assay (108), in which Smaug was used as the bait, confirmed the interaction with NOT3, detectable by cell growth under selective conditions upon co-expression of bait and prey (Figure 3D, left panel). (Note that CAF1 and CAF40 allowed growth under selective conditions in the absence of Smaug, thus no conclusions can be drawn regarding an interaction.) In the two-hybrid experiment, a NOT1 fragment comprising amino acids 752–910 also showed an interaction with Smaug (Figure 3D, right panel). However, this region of NOT1 is not present in the CCR4–NOT_MINI_ complex, which is sufficient for Smaug-dependent deadenylation, whereas it is present in the ^Hs^CCR4–NOT_CORE_ complex, which does not respond to Smaug. Therefore, this region of NOT1 is neither required nor sufficient for a functional interaction with Smaug, and we did not pursue the interaction. Interactions between Smaug and fragments of NOT3 were also examined by means of the two-hybrid assay. The results confirmed the ability of the C-terminal region of NOT3 to associate with Smaug (Figure 3E). The Smaug-NOT3 interaction visible in the ReLo and two-hybrid assays is consistent with the requirement for the NOT module in deadenylation assays (Figure 2D). Mapping of the interaction surface to the C-terminal region of NOT3 is also consistent with the activity of ^Hs^CCR4–NOT_MINI_ in Smaug-dependent deadenylation: In this complex, truncation of NOT3, just upstream of the NOT1-interacting region, leaves the Smaug-interacting region intact (27). In summary, *in vivo* interaction assays and deadenylation assays demonstrate that Smaug interacts with the NOT module of CCR4–NOT, in particular with the C-terminal region of NOT3.

### Both CCR4 and CAF1 contribute to Smaug-dependent deadenylation

**Figure 4.**
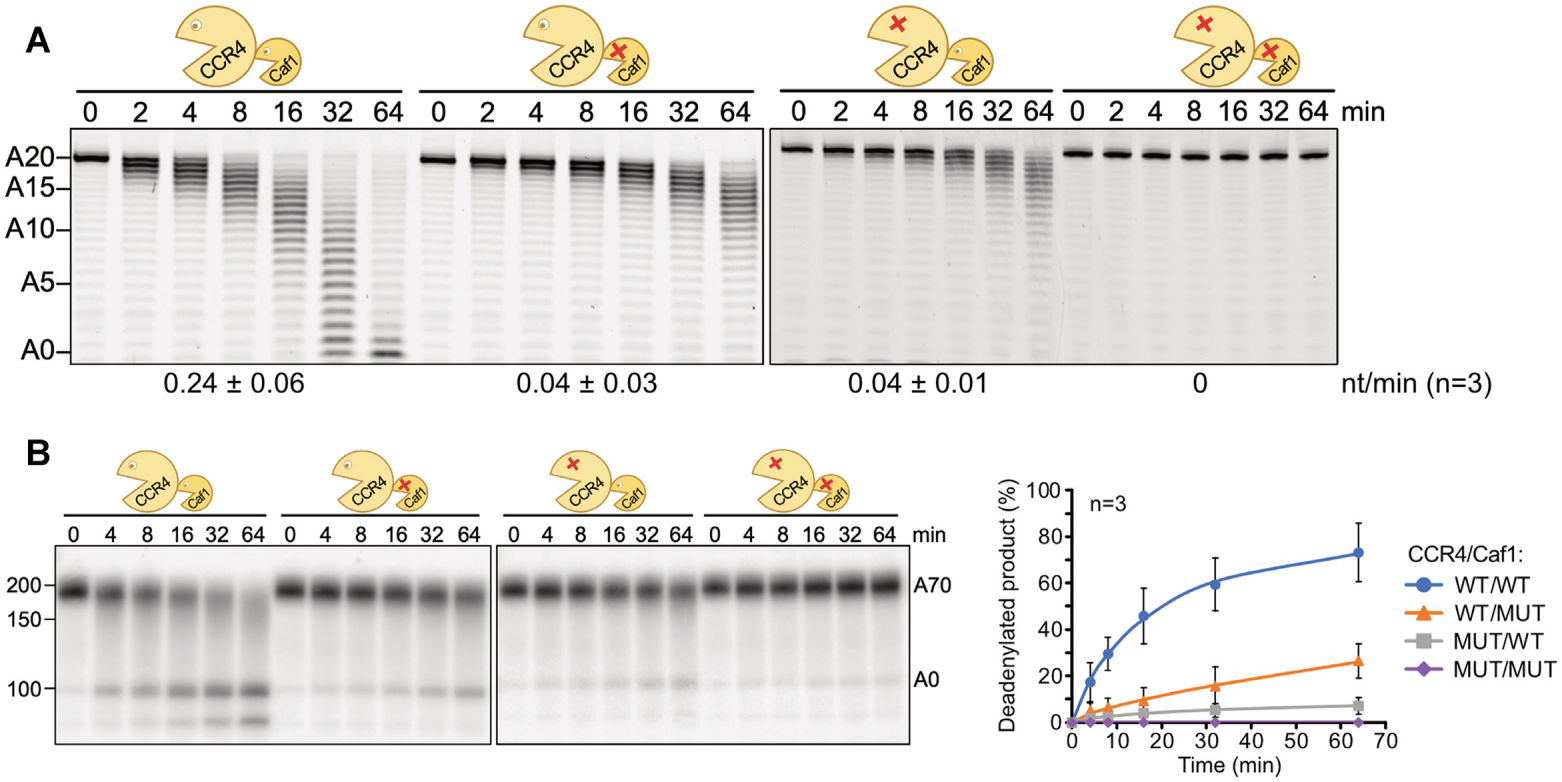
CCR4 and CAF1 make similar contributions to the activity of the ^Dm^CCR4–NOT_MINI_ complex. (**A**) Inactivation of either catalytic subunit has a similar effect on the basal activity of ^Dm^CCR4–NOT_MINI_. Point mutations in the active sites of CCR4 and CAF1 are depicted in the cartoons. 50 nM FAM 7mer-A_20_ RNA was incubated with the respective enzyme complexes (5 nM) for the times indicated. Numbers at the bottom represent average deadenylation rates in nt/min plus/minus standard deviation, based on *n* = 3. (**B**) Inactivation of either catalytic subunit has a similar effect on Smaug-dependent deadenylation. 40 nM Smaug was pre-incubated with 10 nM SRE^WT^-A_70_ substrate RNA, deadenylation was started by the addition of 2 nM ^Dm^CCR4–NOT_MINI_ complex and stopped at the times indicated. The right panel shows a quantification of the fully deadenylated product (average plus/minus standard deviation based on *n* = 3).

Earlier experiments employing RNAi or overexpression of catalytically inactive mutants suggested that CAF1 makes the main contribution to the catalytic activity of the CCR4–NOT complex in *Drosophila* cells (69,109). We used the reconstituted complex to examine the relative importance of CAF1 and CCR4 *in vitro*. ^Dm^CCR4–NOT_MINI_ was prepared with point mutations in the active sites of either nuclease or the combination of both. In assays employing the FAM 7mer-A_20_ RNA as a substrate, the inactivation of either nuclease subunit had an unexpectedly large effect, reducing the activity of the complex by about 80% (Figure 4A). This behavior was seen with several independent preparations of the enzyme complexes and is most likely explained by either inactive subunit exerting a dominant-negative effect on the active subunit, perhaps by transiently blocking access to the 3’ end. An even stronger effect of the same type has been reported for human CCR4–NOT (110). The combination of mutations in both CCR4 and CAF1 abolished the catalytic activity of the complex (Figure 4A). The Smaug-dependent reaction was also impaired by point mutations in either catalytic subunit, and again either single mutant reduced the activity by more than 50%. Combined mutation of both subunits prevented Smaug-dependent deadenylation (Figure 4B). As the inactivation of either catalytic subunit reduced deadenylation activity to a similar extent, both in basal and Smaug-stimulated deadenylation, we conclude that, under our experimental conditions, both subunits make approximately equal contributions to the activity of the CCR4–NOT complex.

**Figure 5.**
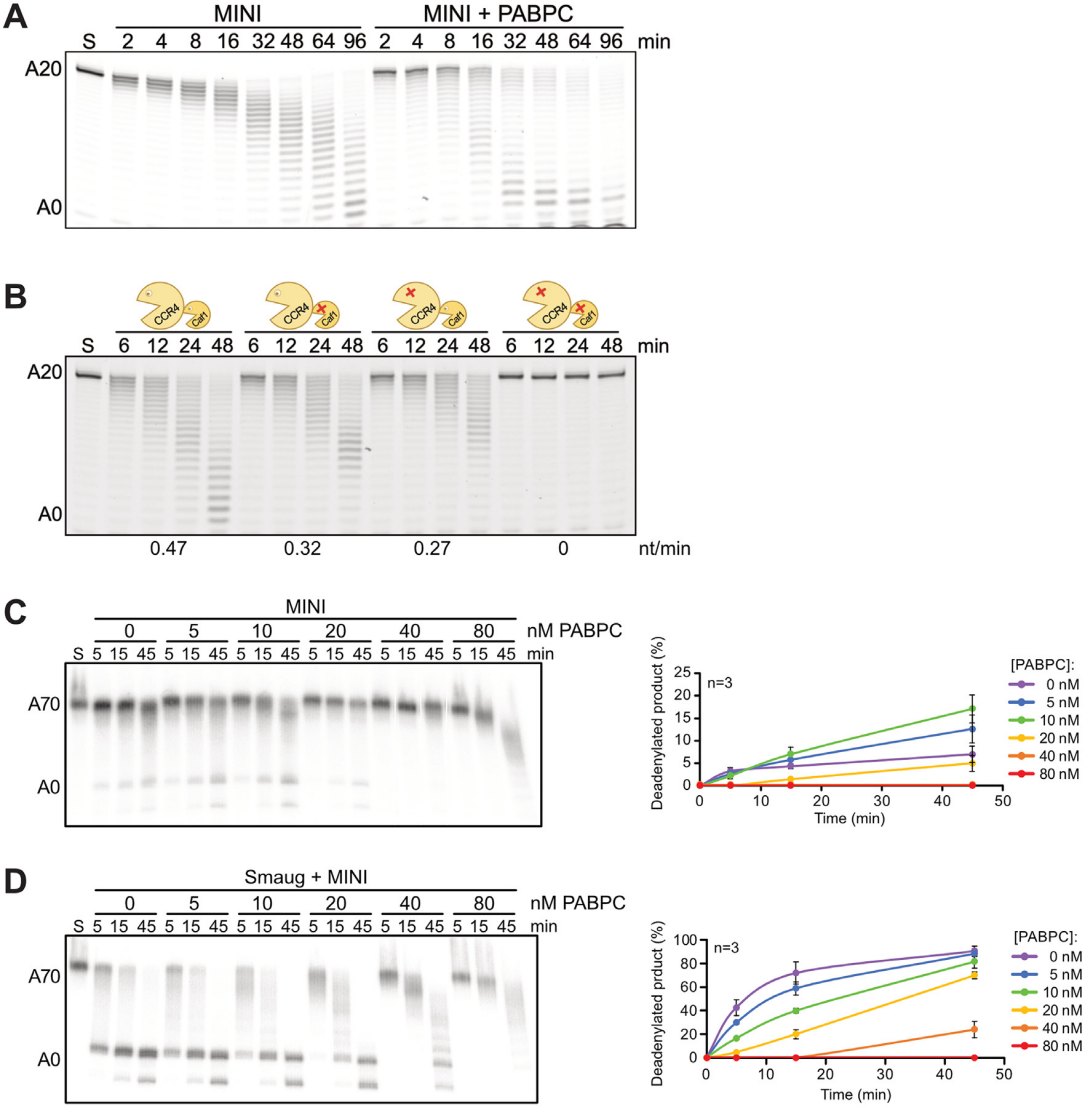
^Dm^CCR4–NOT-dependent deadenylation is moderately affected by PABPC. (**A**) PABPC stimulates the deadenylation of FAM 7mer-A_20_. Substrate RNA (25 nM) was incubated with 200 nM PABPC in the presence of tRNA, and deadenylation was started by the addition of 2.5 nM ^Dm^CCR4–NOT_MINI_. A representative experiment of *n* = 3 is shown. (**B**) Both CCR4 and CAF1 can degrade a poly(A) tail bound by PABPC. Deadenylation time courses were carried out with wild-type ^Dm^CCR4–NOT_MINI_ or mutant variants as indicated. Reaction conditions were as in (**A**). PABPC was present at 200 nM. Numbers at the bottom report deadenylation rates (nt/min). A representative experiment of *n* = 2 is shown. (**C**) PABPC modestly stimulates deadenylation of SREonly-A_70_. SRE^WT^only-A_70_ RNA (5 nM) was deadenylated in the presence of the indicated concentrations of PABPC. ^Dm^CCR4–NOT_MINI_ was used at 0.5 nM. A negative control (S) contained 80 nM PABPC in the absence of CCR4–NOT. The graph shows the accumulation of completely deadenylated product (average plus/minus standard deviation based on (*n* = 3). The curves for 40 and 80 nM PABPC lie on top of each other. (**D**) PABPC inhibits SRE-dependent deadenylation. SRE^WT^only-A_70_ RNA (5 nM) was first pre-incubated with Smaug (30 nM) or buffer for 20 min, then the indicated amounts of PABPC or buffer were added, and the incubation was continued for another 20 min. Finally, deadenylation was started by the addition of ^Dm^CCR4–NOT_MINI_ (0.5 nM). Quantification was as in (**C**).

*In vivo*, the substrate for deadenylation is not naked poly(A), but a complex of poly(A) and the cytoplasmic poly(A) binding protein, PABPC. Moreover, PABPC is part of the SRE-dependent repressor complex (68). The *S. pombe* and human PABPC orthologues stimulate the basal activities of the cognate CCR4–NOT complexes (26,92) but *S. pombe* PABPC was somewhat inhibitory to deadenylation accelerated by the Puf3 protein (44). Thus, we sought to examine the effect of PABPC on deadenylation by *Drosophila* CCR4–NOT. With the FAM 7mer-A_20_ RNA, relatively high concentrations of *Drosophila* PABPC were necessary for complete binding (Supplementary Figure 4A), perhaps because the excess tRNA used as carrier competed for binding, although we cannot exclude partial inactivity of the PABPC preparation. As expected, the protein stimulated deadenylation by ^Dm^CCR4–NOT_MINI_ both at saturating and sub-saturating concentrations. PABPC itself was devoid of deadenylation activity (Supplementary Figure 4B). In a more detailed deadenylation time course, PABPC was initially inhibitory, and the stimulatory effect became apparent only at later times. Size distributions of the RNA were also more heterogeneous in the presence of PABPC (Figure 5A). Presumably, PABPC initially sequesters the 3’ end of the RNA from attack by the nuclease; once the enzyme has gained a foothold on the substrate, PABPC facilitates further deadenylation. Versions of CCR4–NOT carrying inactivating point mutations in either CCR4 or CAF1 responded similarly to PABPC: Both CCR4 and CAF1 were able to degrade poly(A) covered by PABPC, although CCR4 appeared to be slightly more efficient (Figure 5B). The deadenylation of RNAs carrying long poly(A) tails was assayed in the absence of tRNA. Under these conditions, PABPC had again a weak stimulatory effect at low concentrations (5–10 nM; Figure 5C). Native gel electrophoresis suggested that, at 10 nM, about one copy of PABPC was bound per RNA molecule (Figure 5C; Supplementary Figure 4C). Higher concentrations of PABPC (20 – 80 nM) were inhibitory (Figure 5C). Native gel electrophoresis suggested that these concentrations resulted in the binding of up to four copies of PABC (Supplementary Figure 4C). Thus, PABPC binding is likely to extend into the RNA body and may interfere with binding of CCR4–NOT to upstream RNA sequences. In contrast to basal deadenylation, the Smaug-dependent degradation of an A_70_ tail by the CCR4–NOT complex was inhibited by PABPC at any concentration. Whereas inhibition was modest upon binding of about one copy of PABPC, additional PABC inhibited strongly (Figure 5D, Supplementary Figure 4C).

### Smaug makes deadenylation processive

Accessory factors often boost the activity of nucleic acid-polymerizing or -degrading enzymes by increasing their processivity (111,112). A deadenylase can be judged to be processive by two criteria: First, under conditions of excess substrate over enzyme, largely or completely deadenylated products will co-exist with untouched substrate because a processive enzyme will act repeatedly, without dissociation, on the small fraction of substrate to which it is first bound, leaving the rest for later rounds. A completely distributive enzyme, in contrast, will remove single nucleotides from random RNA molecules and thus shorten an excess of substrates in a synchronous manner through multiple rounds of association and dissociation. Second, if a poly(A) tail is completely degraded without intermittent dissociation of the enzyme, the rate at which an individual poly(A) tail is shortened will be independent of the concentration of the processive nuclease or its ratio to substrate. Thus, the time at which the deadenylation end product is first seen will be independent of the nuclease concentration; only the amount of end product present at this time will increase with increasing nuclease concentration. In contrast, for a distributive enzyme the time required for the deadenylation end product to appear will be shorter with higher enzyme concentrations. This is because a high enzyme concentration will drive the association with substrate preceding every catalytic event.

**Figure 6.**
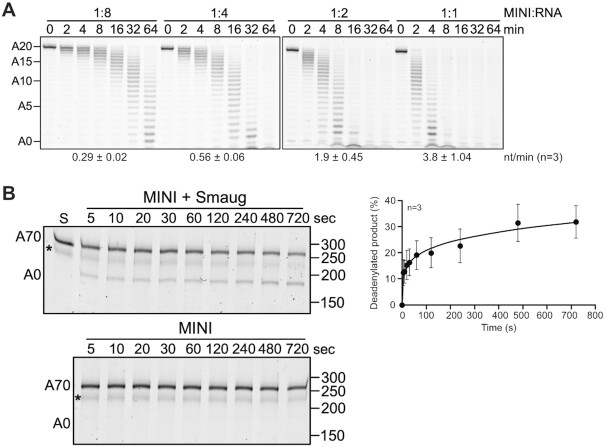
Smaug makes the CCR4–NOT complex processive. (**A**) The ^Dm^CCR4–NOT_MINI_ complex is distributive on its own. A constant concentration of the FAM 7mer-A_20_ RNA (50 nM) was incubated with 6.25, 12.5, 25 or 50 nM ^Dm^CCR4–NOT_MINI_, resulting in the molar ratios indicated. Aliquots were withdrawn at the time points indicated. Numbers at the bottom represent average deadenylation rates in nt/min plus/minus standard deviation, based on *n* = 3. (**B**) The ^Dm^CCR4–NOT_MINI_ complex acts processively in Smaug-dependent deadenylation. The substrate RNA in this assay was fluorescently labelled TCE^WT^-A_70_ RNA, which was used at 50 nM. The RNA was pre-incubated with or without 300 nM Smaug as indicated and the reaction started by the addition of ^Dm^CCR4–NOT_MINI_ (5 nM). Aliquots were withdrawn as indicated. Note that the time scale is in seconds. The asterisk indicates an unknown RNA species that we have not been able to remove. The graph on the right shows the accumulation of fully deadenylated product in the reaction containing Smaug (average plus/minus standard deviation based on *n* = 3)

The processivity of the CCR4–NOT complex by itself was first assessed in reactions with the FAM 7mer-A_20_ RNA. As shown in Figure 6A, fully deadenylated products did not co-exist with untouched substrate and became visible only at later time points when all of the substrate had already been shortened to a significant extent. Also, fully deadenylated product first became visible at earlier time points in proportion with increasing enzyme concentration (e. g. ∼32 min at a 1:8 ratio of CCR4–NOT:RNA versus 4 min at a 1:2 ratio); in other words, the rate of shortening of an individual poly(A) tail was dependent on the nuclease concentration. By both criteria, the activity of CCR4–NOT with this substrate was distributive.

To determine whether Smaug-dependent deadenylation is processive, we pre-incubated SRE-containing TCE^WT^ RNA (see Materials and Methods) with a threefold excess of Smaug over SREs. Limiting amounts of *^Dm^*CCR4–NOT_MINI_ were then added, and time-dependent deadenylation was measured. Biphasic kinetics were observed: In an initial burst phase, fully deadenylated end product was already present at the first time point, only five seconds after the start of the reaction. At this early time point, most of the substrate RNA had not been attacked, and partially shortened intermediates of deadenylation were not visible. Thus, the reaction was processive. In the second phase of the reaction, additional deadenylation product accumulated at a decreasing and overall much lower rate (Figure 6B). Since the initial reaction was so fast, we could not test the prediction that the time at which the end product first appeared should be independent of enzyme concentration. However, the amount of RNA that was completely deadenylated within the burst phase was approximately stoichiometric with the CCR4–NOT complex: 5 nM CCR4–NOT complex deadenylated approximately 12% of 50 nM substrate RNA within 5 s. Thus, the initial burst phase represented the complete deadenylation of approximately one substrate RNA per enzyme complex, whereas the subsequent slow increase in deadenylated RNA presumably reflected the rate-limiting transition of the enzyme to new substrate molecules. In the control reaction lacking Smaug, no deadenylation was detectable under these conditions (Figure 6B), but weak distributive activity was visible with longer incubation times (Supplementary Figure 5).

### Cup also induces deadenylation

**Figure 7.**
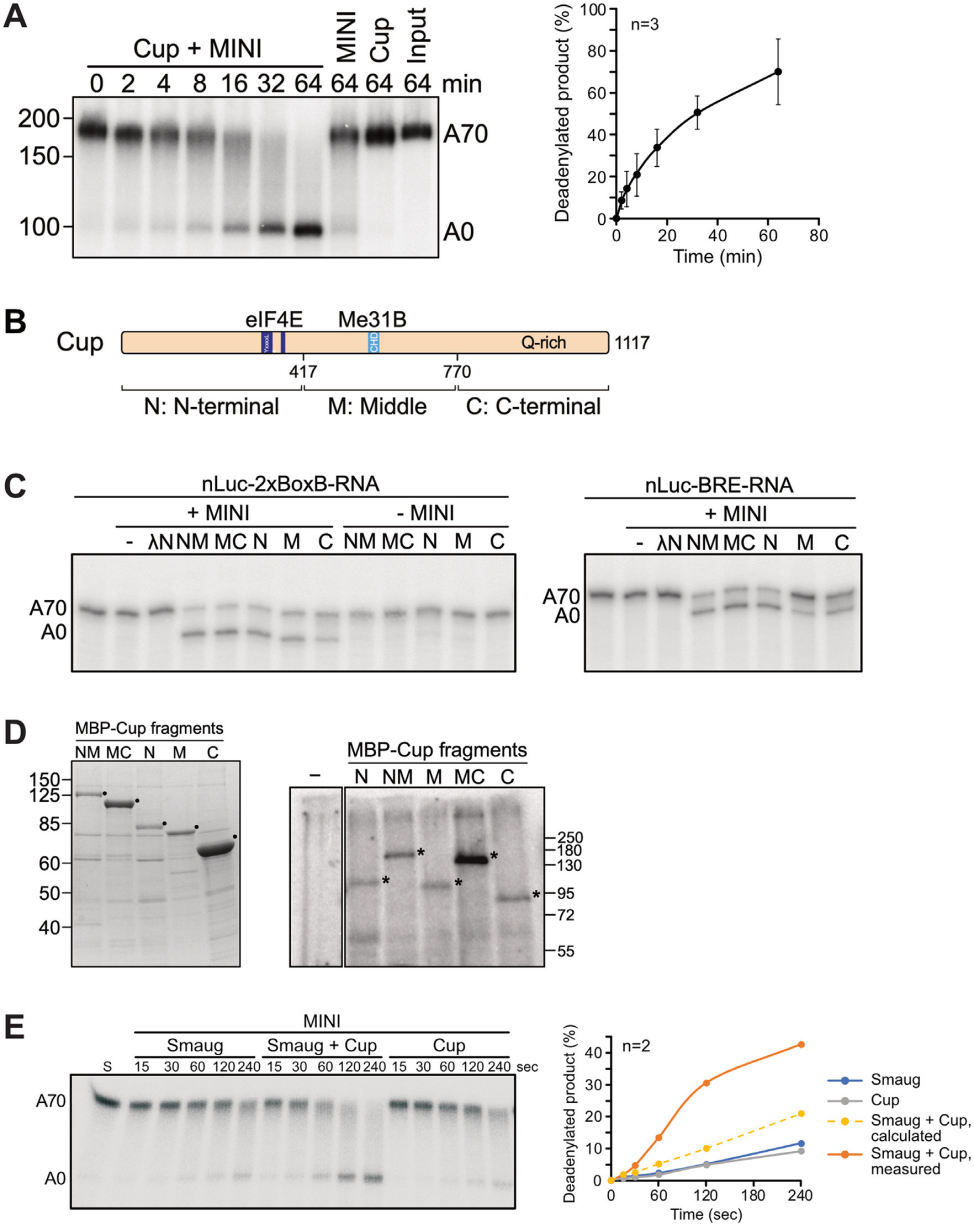
Cup also induces deadenylation. (**A**) Cup induces deadenylation by the ^Dm^CCR4–NOT_MINI_ complex. SRE^WT^-A_70_ RNA (20 nM) was pre-incubated with 80 nM Cup, and deadenylation was initiated by the addition of 2 nM ^Dm^CCR4–NOT_MINI_. Aliquots were withdrawn at different time points as indicated. Controls included incubations in the absence of Cup, with Cup only, or no protein added. The graph shows the accumulation of completely deadenylated product in the Cup + CCR4–NOT reaction (average plus/minus standard deviation based on *n* = 3). (**B**) A scheme of Cup showing its division into an N-terminal part harbouring the eIF4E binding motifs, a middle part with the Cup homology domain (CHD) binding Me31B, and a C-terminal part rich in glutamine residues. (**C**) The ability of Cup to stimulate the CCR4–NOT complex is distributed over the protein. The Cup fragments shown in (A) were fused to His-λN-MBP and purified (Supplementary Figure 7B). Proteins (40 nM each, except M, which was 80 nM) were pre-incubated for 15 min either with the nLuc-2xBoxB-A_70_ RNA (2 nM; left panel) or with the nLuc-BRE^MUT^-A_70_ RNA (2 nM, right panel). Deadenylation was started by the addition of ^Dm^CCR4–NOT_MINI_ (1 nM) and allowed to proceed for 30 min. Controls included incubations in the absence of CCR4–NOT, with CCR4–NOT only or with CCR4–NOT plus His_8_-λN-MBP. A representative experiment of *n* = 2 is shown. (**D**) Cup and all its fragments can be UV-crosslinked to RNA. Left panel: Coomassie-stained SDS gel showing MBP-Cup fragments lacking λN. Right panel: Cup fragments shown in the left panel were UV-cross-linked to radiolabeled RNA. Cross-linking products were analyzed by SDS-polyacrylamide gel electrophoresis and autoradiography. (**E**) Smaug and Cup jointly stimulate deadenylation. Reactions were carried out in the absence of PEG. The SRE^WT^-A_70_ RNA (10 nM) was pre-incubated with Smaug (80 nM) or with buffer, then Cup (80 nM) or buffer was added for an additional 20 min. Deadenylation was started by the addition of ^Dm^CCR4–NOT_MINI_ (1 nM). Left panel: Analysis of reaction products by denaturing gel electrophoresis. Right panel: Completely deadenylated products were quantified (average of *n* = 2). The broken yellow line indicates theoretical product accumulation predicted by additive behavior of Smaug and Cup.

Additional constituents of the Smg-dependent repressor complex (Cup, Me31B, Tral, Belle) were also titrated into deadenylation assays containing the CCR4–NOT complex. Me31B was inactive in the presence or absence of ATP (Supplementary Figure 6). Tral and Belle were also inactive, and so was a combination of Tral and Me31B (data not shown). However, Cup consistently stimulated deadenylation (Figure 7A). Thus, the Cup-dependent stimulation of deadenylation reported by Igreja and Izaurralde (78) is a direct effect. MINI and FULL complexes were similar in their response to Cup (Supplementary Figure 1C). Like Smaug-dependent deadenylation, the Cup-dependent reaction was also strongly stimulated by PEG (Supplementary Figure 2B). As expected, Cup-dependent deadenylation was not SRE-dependent (Supplementary Figure 6A). We separated Cup into three non-overlapping fragments, the N-terminal, middle and C-terminal domains (N, M and C) (78). The three fragments as well as the NM and MC combinations were initially produced as His-λN-MBP fusion proteins (Figure 7B, Supplementary Figure 7B). The phage λ N peptide (98) allowed binding to a deadenylation substrate containing two box B elements 16 nucleotides upstream of an A_70_ tail. All Cup fragments prepared in this manner were able to stimulate deadenylation of the box B-containing substrate (Figure 7C, left panel). No deadenylation was visible when Cup fragments were replaced by His-λN-MBP as a control, and the Cup fragments had no activity on their own. The ability of the M and C fragments to stimulate deadenylation is in agreement with the results of Igreja and Izaurralde obtained in cells (78). Surprisingly, all Cup fragments also stimulated deadenylation of a substrate RNA in which the box B elements were replaced by a control sequence (Figure 7C, right panel); only the M fragment had weaker activity with this substrate.

The activity of wild-type Cup and the box B-independent activity of the Cup fragments suggested that the protein and its fragments can bind RNA and recruit the CCR4–NOT complex. In fact, Cup has been identified as an RNA binding protein by proteome-wide UV cross-linking screens (113,114), and so has the related protein 4E-T in mammals (115). Unfortunately, purified Cup and its fragments did not yield interpretable results in electrophoretic mobility shift experiments and were not clean enough for nitrocellulose filter-binding experiments. Thus, UV cross-linking to a radiolabeled synthetic RNA oligonucleotide was used to assess the ability of Cup to bind RNA. For these experiments, use of the His-λN-MBP tag was avoided because of the RNA binding activity of the N peptide. Instead, all five Cup fragments were purified as MBP fusion proteins from baculovirus-infected Sf21 cells (Figure 7D). All these Cup fragments promoted deadenylation, albeit weakly (Supplementary Figure 7C), and all could be cross-linked to RNA (Figure 7D). As controls, the MBP tag alone and BSA were not cross-linked under the same conditions (Supplementary Figure 7D). Migration of the major cross-link products in the SDS gel varied as expected from the molecular weights of the Cup variants, providing evidence that the signals were in fact derived from Cup. In order to further examine the identities of the cross-link products, we generated His-MBP fusions of full-length Cup (in Sf21 cells) and of the M, MC and C fragments (in *E. coli*). We were unable to generate the N and NM fragments with this type of fusion. Fusion proteins were cross-linked to RNA, and their identities were ascertained by their binding, under denaturing conditions, to a Ni-NTA matrix (Supplementary Figure 7E). Some proteolytic products common to the M and MC fragments were co-purified on the Ni-NTA column, but a control protein, GST-Tral, was not, confirming the specificity of the pull-down. Thus, full-length Cup as well as all fragments tested are able to bind RNA.

We wished to test whether Smg and Cup can simultaneously stimulate CCR4–NOT-dependent deadenylation. However, under standard reaction conditions, Smaug-dependent deadenylation was so efficient (Figure 6) that no further stimulation by Cup would have been detectable. Thus, the reaction was weakened by omission of PEG from the buffer. Under these sensitized conditions, the simultaneous presence of Smaug and Cup indeed led to significantly improved deadenylation of the SRE^WT^ RNA compared to either protein alone; stimulatory effects were reproducibly more than additive (Figure 7E). Each protein was used at a concentration that individually was saturating for deadenylation and close to saturation in RNA binding (Supplementary Figure 7F, G). Thus, Smg and Cup bound to the same RNA can cooperate in the stimulation of the CCR4–NOT complex.

## DISCUSSION

During early embryonic development of *Drosophila*, Smaug is responsible for the degradation of hundreds of maternal mRNAs, thus making a major contribution to the maternal-to-zygotic transition (65,116). Its best-studied target is the *nanos* mRNA. Smaug represses *nos* both by preventing its translation and by inducing its deadenylation by the CCR4–NOT complex. As Smaug binds the *nos* 3’-UTR in the company of six other proteins, the mechanism by which it induces deadenylation is potentially complex. In order to examine the mechanism of the deadenylation reaction, we have reconstituted the eight subunit *Drosophila* CCR4–NOT complex and also overproduced and purified Smaug as well as the other constituents of the Smaug-dependent repressor complex. Activity assays with these proteins revealed that Smaug on its own is able to induce an efficient, processive deadenylation by the CCR4–NOT complex. As a second component of the repressor complex, Cup was also able to stimulate CCR4–NOT-catalyzed deadenylation. A third component, PABPC, modestly promoted basal deadenylation, but was moderately inhibitory to Smaug-dependent deadenylation. The other components of the repressor complex did not affect deadenylation in our *in vitro* assays. The inactive components included Me31B, even though the protein is able to bind NOT1 (33,34,82).

Our data support a simple tethering model for the ability of Smaug to accelerate deadenylation: Smaug binds SREs with high affinity (63,117). As shown here, the protein also interacts directly with the CCR4–NOT complex via the C-terminal domain of NOT3 and induces a processive activity of CCR4–NOT. Smaug-induced deadenylation proceeded at a rate too fast to be measured by manual pipetting. The amount of RNA deadenylated in the initial burst appeared to be stoichiometric with respect to the deadenylase, although this conclusion is limited by the accuracy with which the concentration of CCR4–NOT could be determined. These data indicate that Smaug promotes deadenylation by preventing the dissociation of the deadenylase from its substrate. This corresponds to the mechanism by which deadenylation effectors Mmi1, Puf3 and Zfs1 from *S. pombe* stimulate their cognate CCR4–NOT complex (37,44). Smaug-dependent deadenylation was initially speculated to be more complex, as the reaction was sensitive to ATP depletion in *Drosophila* embryo extracts (72). Deadenylation induced by miRNAs behaved similarly (118). However, the apparent ATP requirement for miRNA-dependent deadenylation was later explained by the accumulation of AMP, presumably derived from ADP by the reversible adenylate kinase reaction; AMP inhibited deadenylation (119). We have independently found that the apparent ATP-dependence of deadenylation in embryo extract was only observed with substrate RNAs produced by run-off transcription that ended in a few non-A residues downstream of the poly(A) tail due to the restriction site used at the time to linearize the template; deadenylation was not inhibited by ATP depletion when the poly(A) tails were added by poly(A) polymerase (C. Temme and EW, unpublished data). Perhaps degradation of the non-A residues is more sensitive to AMP inhibition. Regardless, our reconstitution experiments confirm that Smaug-dependent deadenylation does not require ATP.

Deadenylation effectors typically interact with CCR4–NOT in a complex manner, employing multiple short interaction motifs embedded in intrinsically disordered regions (37,44,120). For example, contacts to CCR4–NOT involve four domains of the *Drosophila* Pumilio protein (109) or multiple tryptophan-containing motifs of the miRNA-associated GW182 proteins (33,34,39). Many effectors also interact with more than one subunit of CCR4–NOT. For example, the *Drosophila* Nanos protein is itself an effector of deadenylation and uses redundant binding sites to recruit CCR4–NOT: The dominant site forms one short α-helix contacting NOT1 in its C-terminal domain and a second α-helix binding the NOT box of NOT3 (121). Likewise, *Drosophila* Roquin has a binding site for CAF40 and redundant binding sites for the NOT module (51), and mammalian TTP interacts both with NOT1 (43) and CAF40 (46). In the case of Smaug, the C-terminal region of NOT3 provides the major interaction surface, but our data do not exclude additional interactions. Although the ReLo and two-hybrid assays were performed in the presence of all cellular proteins, both the Smaug – CCR4–NOT interaction in gel filtration and the stimulation of deadenylation by Smaug were observed with highly purified preparations. Thus, the interaction between Smaug and NOT3 is almost certainly direct. As in the case of other deadenylation effectors, multiple regions of Smaug appear to be involved as our attempts to map an individual NOT3 binding domain in the protein were not successful.

In addition to Smaug, Cup was also able, on its own, to stimulate CCR4–NOT-dependent deadenylation. The ability to induce deadenylation seems to be distributed over much of the protein, as each of the three non-overlapping fragments was active. Our results are consistent with earlier results (78) that the M and C fragments of Cup associate with CCR4–NOT and, when tethered to a reporter RNA, induce deadenylation in cells. Our results in the reconstituted *in vitro* system indicate that this effect of Cup is direct. The ability of Cup and its fragments to induce deadenylation even in the absence of tethering suggests they are able to bind RNA. Unfortunately, we have been unable to purify Cup or its fragments to homogeneity, as the proteins did not elute in clean peaks from any column tested. Thus, RNA binding activity in nitrocellulose filter-binding assays could not be attributed to Cup as opposed to contaminations. Upon electrophoresis in native gels, all complexes formed between RNA and the Cup preparation remained stuck in the wells so that, again, the protein responsible for binding could not be identified with certainty. However, UV cross-linking assays were consistent with RNA binding of Cup and its fragments. We have also tested whether Cup interacts with CCR4–NOT. Whereas CCR4–NOT formed a well-defined peak in the included volume of a gel filtration column (Figure 1B), Cup eluted in the void volume, indicating that it is aggregated and/or assumes a large volume due to its intrinsically disordered nature. When Cup and CCR4–NOT were analyzed in combination, both eluted in the void volume (data not shown). While this is evidence of an interaction, the specificity is questionable in view of the disordered and presumably aggregated state of Cup.

Although Cup is able to bind RNA and induce deadenylation on its own, its *in vivo* activity probably depends on the protein being recruited to specific mRNAs by Smaug (75) and other RNA binding proteins, for example Bruno (74,76). The presence of two deadenylation effectors, Smaug and Cup, in a single complex is not without precedent: Nanos and Pumilio and, in some cases, Brat, cooperate in the regulation of *Drosophila hunchback* and other mRNAs (122,123). Both Nanos and Pumilio can individually elicit deadenylation by CCR4–NOT (see above) (109,121,124). When both Smaug and Cup were used at near-saturating concentrations, their effects on deadenylation were not merely additive, but a modest degree of cooperativity was reproducibly observed. Cooperativity is not altogether surprising, as Smaug and Cup are thought to interact directly (75). The cooperative effect was seen in the absence of a crowding reagent; in the presence of PEG, the Smaug-dependent reaction is so efficient that one cannot expect an additional stimulation by Cup.

Previous knock-down experiments and overexpression of catalytically dead polypeptides in cultured *Drosophila* cells suggested that CAF1 may be the dominant catalytic subunit of CCR4–NOT *in vivo* (69,109). The interpretation of these experiments was limited by incomplete protein depletion and an uncertain degree to which WT protein was replaced by the inactive version in the CCR4–NOT complex. CCR4 is important for the deadenylation of *nos* (67) (see below), and genetic evidence for the importance of CCR4 catalytic activity has been presented (123). Our reconstitution experiments now provide clear evidence that both CCR4 and CAF1 are active nucleases. Individual inactivation of either subunit reduced the activity of the complex by more than 50%. This apparent interdependence of the two activities may be most easily explained by the inactive subunit transiently blocking access to the 3’ end. Nevertheless, inactivation of either CCR4 or CAF1 reduced the activity of the complex to a similar extent, both in unassisted, basal deadenylation and in the Smaug-dependent reaction, suggesting comparable contributions of the two nucleases to the activity of CCR4–NOT. However, since the activity of CAF1 is highly sensitive to pH and the concentrations of Mg^2+^ and Zn^2+^ (31), our results do not exclude the possibility that one catalytic subunit plays a dominant role *in vivo*.

Using a fully reconstituted CCR4–NOT complex from *S. pombe*, Webster et al. (92) found that the enzyme's basal activity is facilitated by PABPC (Pab1 in *S. pombe*). Similar conclusions have been reached for the human deadenylase (26), although reconstitution was limited to a CCR4-CAF1 heterodimer. In accordance with these data, we also observed that the basal deadenylation activity of the *Drosophila* CCR4–NOT complex was moderately enhanced by PABPC at appropriate concentrations. In contrast, when added to a Smaug-containing deadenylation reaction, PABPC caused a modest inhibition; excess protein was strongly inhibitory. Webster et al. (44) also reported a weak inhibitory effect of PABPC on the activity of *S. pombe* CCR4–NOT activated by an RNA binding protein. In summary, PABPC can be described as being largely permissive for specific deadenylation, i.e. the strong effects of the deadenylation effectors that have been tested are dominant compared to the modest effects of PABPC.

Webster *et al.* (92) and Yi *et al.* (26) found that the catalytic activity of *S. pombe* or human CCR4 was stimulated by PABPC, whereas CAF1 was unable to degrade PABPC-bound poly(A). In contrast, we observed that both catalytic subunits of *Drosophila* CCR4–NOT are able to cope with a PABPC-poly(A) complex. This discrepancy may reflect species-specific differences. However, Stupfler *et al.* (125) reported that mouse CAF1 (CNOT7) digested PABPC-bound poly(A) and the naked polymer with similar efficiencies; thus, it seems more likely that the effect of PABPC depends on the reaction conditions, to which CAF1 is highly sensitive. Mice lacking both CCR4 orthologues (CNOT6 and CNOT6L) were viable, had no overt phenotype, and fibroblasts derived from them had a normal poly(A) tail length distribution. In contrast, the deletion of NOT1 or combined deletion of both CAF1 orthologues (CNOT7 and CNOT8) were lethal, and their reduced expression led to an accumulation of long poly(A) tails (126). These data show that CCR4–NOT is essential, but the catalytic activities of CCR4-type subunits are not. Thus, CAF1-type subunits must be able to degrade PABPC-covered poly(A) *in vivo*; any impediment imposed by PABPC cannot be absolute. The ability of CAF1 to degrade poly(A) in the presence of PABPC is also enhanced in the presence of BTG2/TOB (38,125,127).

Our data reveal that Cup plays a direct role in the deadenylation of *nos* and probably many other RNAs. Thus Smaug, acting on CCR4–NOT by itself and promoting the recruitment of Cup (75), accelerates deadenylation directly and presumably also indirectly. However, the experiments capture only select aspects of the complicated *in vivo* situation: Smaug and its partner Cup are not the only effectors of *nos* deadenylation; piRNAs are also involved (128), and accordingly a deletion of Smaug only partially stabilizes *nos* RNA (129). Whereas *nos* loses its entire poly(A) tail in the Smaug- and CCR4–NOT-dependent *in vitro* reaction, the RNA persists with an oligo(A) tail *in vivo* (see Introduction). Possibly, the complete and extremely rapid deadenylation reaction observed *in vitro* is tempered by conditions prevailing in the embryo. However, a competing poly(A) tail extension reaction catalyzed by the non-canonical poly(A) polymerase GLD2 (encoded by *wispy*) presumably plays a more important role (130).

In the early *Drosophila* embryo, translational efficiency is tied to poly(A) tail length (21). Therefore, Smaug-induced deadenylation, even though it does not go to completion in the embryo, contributes to the translational repression of the non-localized fraction of *nos* RNA (67). However, *nos* translation is also repressed by mechanisms independent of deadenylation (68,73,75). Although CCR4–NOT can repress translation independently of its deadenylation activity (39,40,86,131), the deadenylase is an unstable and/or substoichiometric constituent of the Smaug-dependent repressor complex (68) and therefore unlikely to play a major role in deadenylation-independent repression.

Deadenylation not only contributes to translational repression, but is also the first step in the decay of *nos*, as shown by the stabilization of *nos* in *smg* and *twin* mutants (67). Cup stabilizes deadenylated RNA by inhibiting decapping (78). Thus, the degradation of Cup during the first three hours of embryonic development (132) might be thought to be essential for the further degradation of deadenylated *nos*. However, *nos* is cleared rapidly and completely in *wispy* mutants (130); thus, even in the presence of Cup, deadenylation appears to be sufficient to induce the decay of the *nos* mRNA.

## DATA AVAILABILITY

All data are incorporated into the article and its online supplementary material.

## Supplementary Material

gkad159_Supplemental_File
