# Supplementary material for: RNA binding proteins Smaug and Cup induce CCR4–NOT-dependent deadenylation of the nanos mRNA in a reconstituted system
Source: Nucleic Acids Res. 2023 Mar 23;51(8):3950–70. doi: 10.1093/nar/gkad159 (PMC10164591; doi:10.1093/nar/gkad159)
Supplement: gkad159_Supplemental_File [file gkad159_supplemental_file.pdf]

Supplement to

RNA binding proteins Smaug and Cup induce CCR4-NOT-dependent deadenylation of the *nanos* mRNA in a reconstituted system

Filip Pekovic, Christiane Rammelt, Jana Kubíková, Jutta Metz, Mandy Jeske, Elmar Wahle

Inventory:

Supplemental Figure 1
Supplemental Figure 2
Supplemental Figure 3
Supplemental Figure 4
Supplemental Figure 5
Supplemental Figure 6
Supplemental Figure 7
Supplemental Table 1
Supplemental Table 2
Supplemental Table 3

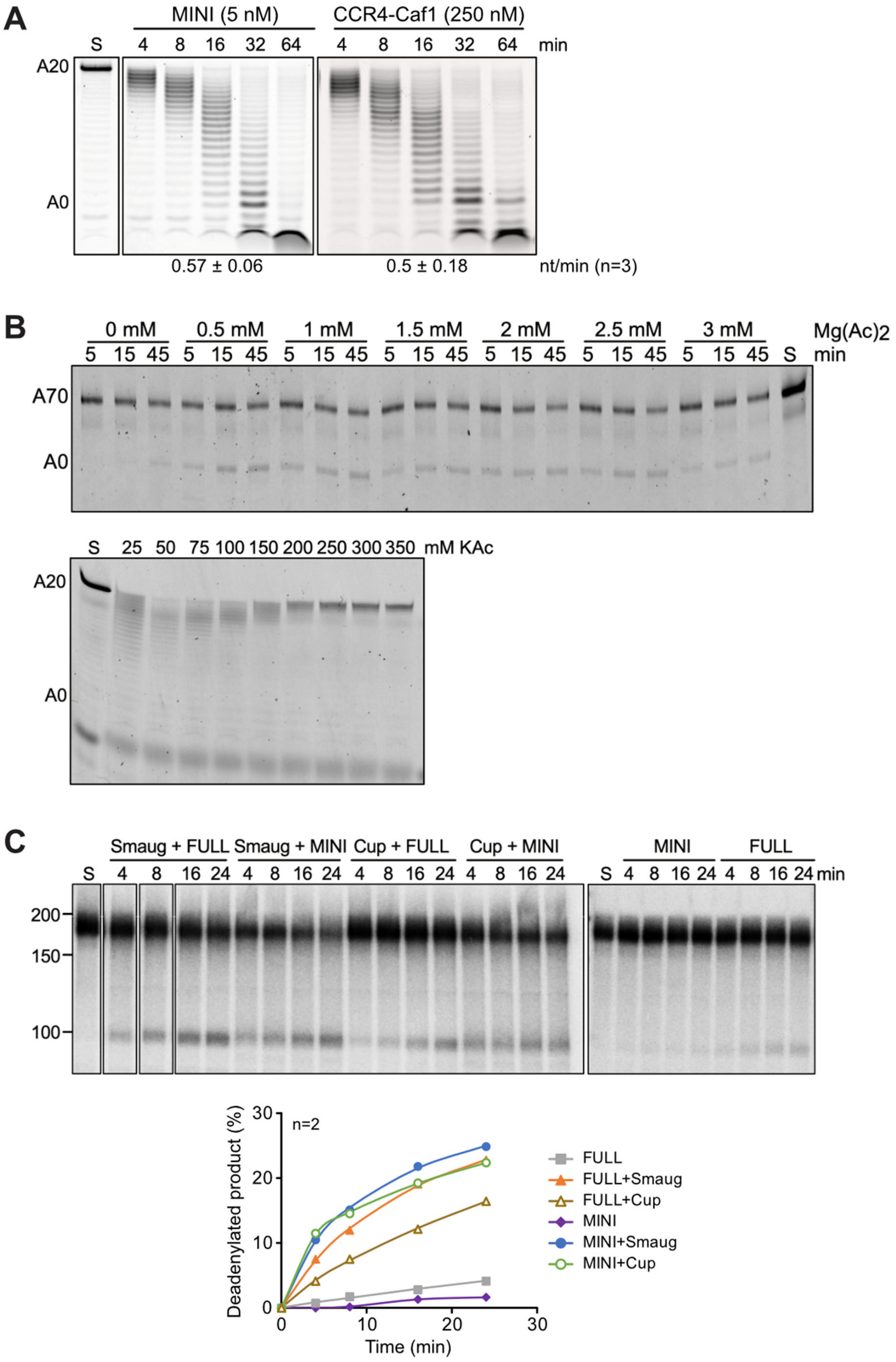

Supplemental Figure 1. Activity of CCR4 and CAF1 depends on other subunits of CCR4-NOT and on ionic conditions

Supplemental Figure 1 (continued). Activity of CCR4 and CAF1 depends on other subunits of CCR4-NOT and on ionic conditions

(A) Deadenylation activity of the CCR4-Caf1 heterodimer is enhanced by other subunits of the CCR4-NOT complex. 50 nM FAM-7mer-A_{20} RNA was incubated with either 5 nM of ^{Dm}CCR4-NOT_{MINI} or 250 nM of ^{Dm}CCR4-Caf1, and aliquots were withdrawn as indicated. Numbers at the bottom represent average deadenylation rates in nt/min plus/minus standard deviation, based on n=3. At the 50fold higher concentration, CCR4-Caf1 shows activity comparable to that of the CCR4-NOT_{MINI} complex.

(B) Deadenylation activity of ^{Dm}CCR4-NOT complex is sensitive to Mg^{2+} and K^{+} concentrations. Top panel: 25 nM FAM-TCE^{MUT}-A_{70} RNA was incubated with 25 nM of ^{Dm}CCR4-NOT_{MINI} in the presence of varying concentrations of magnesium acetate. Aliquots were withdrawn and analyzed at the time points indicated. In this assay, relatively high concentrations of enzyme were used as stability of the CCR4-NOT complex had not yet been optimized. Bottom panel: 10 nM FAM-7mer-A_{20} RNA was incubated with 5 nM of ^{Dm}CCR4-NOT_{MINI} in the presence of increasing concentrations of potassium acetate. These assays were done in the absence of BSA. Deadenylation was stopped after 60 minutes. In addition to the varying concentrations of potassium acetate, the reactions contained 3 mM NaCl introduced with the protein buffer. The strong diffuse band visible at the bottom is bromophenol blue.

(C) ^{Dm}CCR4-NOT_{FULL} and ^{Dm}CCR4-NOT_{MINI} behave similarly in Smaug- or Cup-dependent deadenylation. Reactions contained 20 nM SRE^{WT}-A_{70} RNA, 1 nM of the CCR4-NOT complex indicated and 80 nM Smaug or Cup where indicated. Aliquots were withdrawn and analyzed at the time points indicated. S, unreacted substrate. The graph shows the accumulation of fully deadenylated RNA (average of n=2).

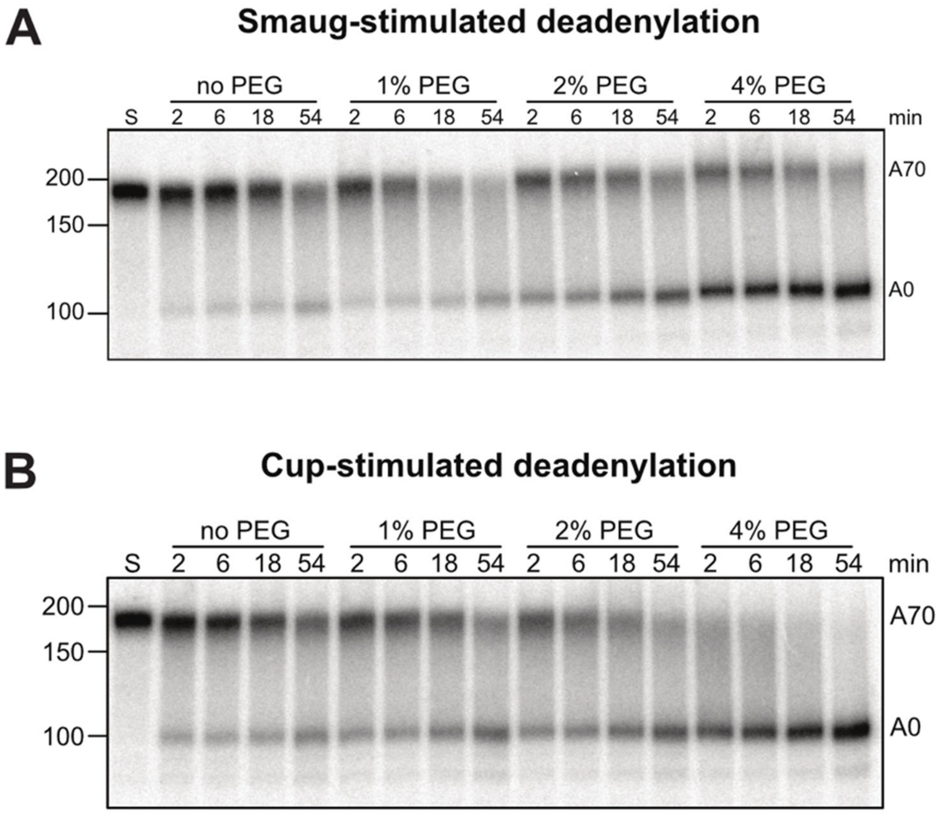

Supplemental Figure 2. Smaug- and Cup-dependent deadenylation is enhanced by a crowding reagent

(A) Smaug-dependent deadenylation is stimulated by a crowding reagent. Deadenylation time courses were carried out with 10 nM SRE^{WT}-A_{70} RNA, 80 nM Smaug and 2 nM ^{Dm}CCR4-NOT_{MINI} in the presence of different concentrations of PEG 20,000.

(B) Cup-dependent deadenylation is stimulated by a crowding reagent. Deadenylation time courses were carried out as in (A) except that 80 nM Cup replaced Smaug.

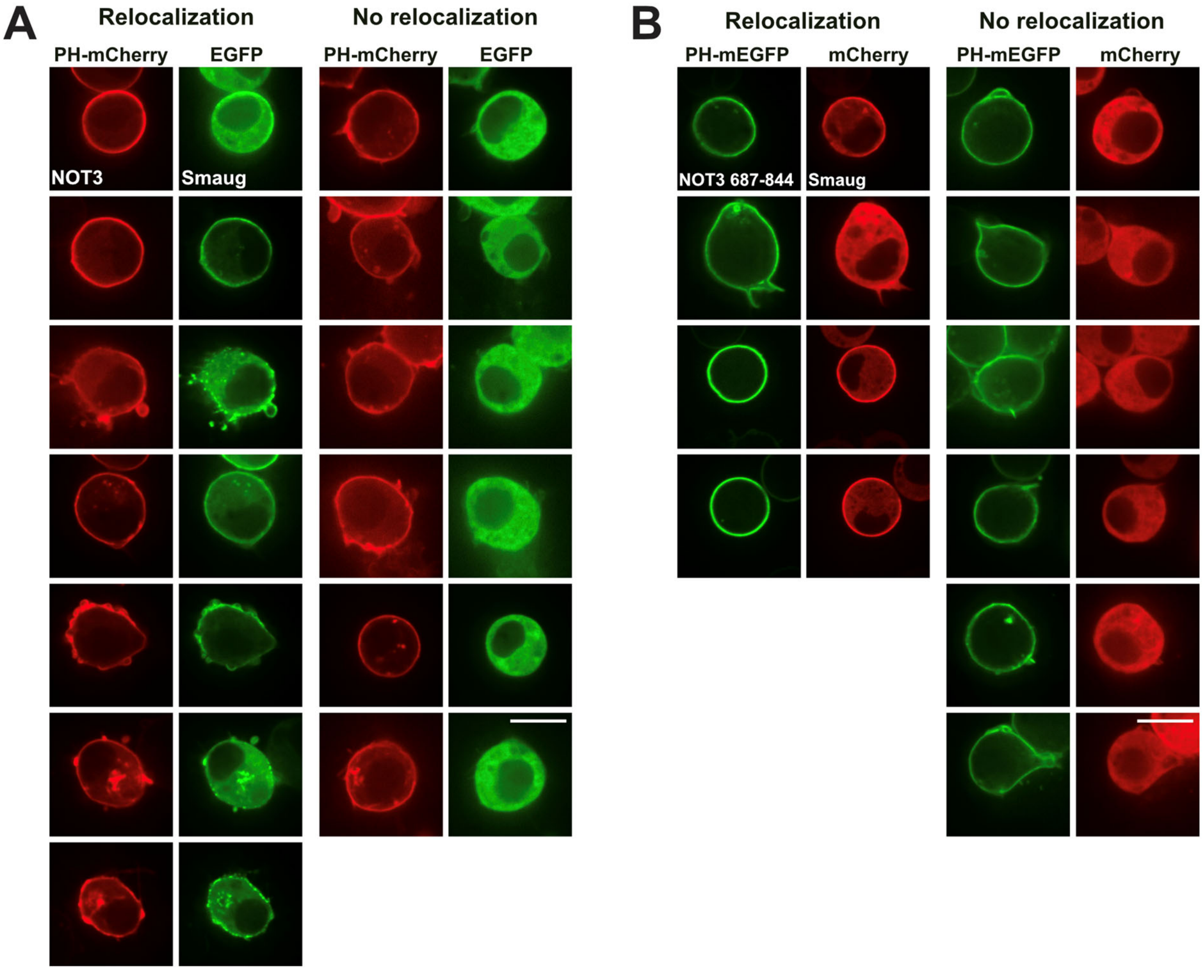

Supplemental Figure 3. Assessment of the Smaug - NOT3 interaction using ReLo assays

(A) NOT3 was fused with PH-mCherry and coexpressed with Smaug fused to EGFP (see **Fig. 3** of main text). The left panel shows a selection of cells in which Smaug relocalized to the plasma membrane. The right panel shows cells in which a relocalization is not obvious.

(B) A C-terminal fragment (aa 687-844) of NOT3 was fused with PH-mEGFP and co-expressed with Smaug fused to mCherry. The left panel presents a selection of cells in which Smaug relocalized to the plasma membrane. Cells in the right panel show no obvious relocalization.

Selections of cells are representative for the entire sample. In contrast to the many examples that we have analyzed and presented previously (Salgania et al. 2022), we experienced difficulties when analyzing the Smaug - NOT3 interaction with ReLo. Coexpression of Smaug and NOT3 caused a reduced cell viability, and the Smaug - NOT3 interaction apears to be weak. Hence, we observed relocalization mainly in cells with high expression levels, but not in cells with low fluorescence signals. Importantly, relocalization of Smaug was only seen with NOT3 and never when Smaug was combined with controls or with other subunits of the CCR4-NOT complex.

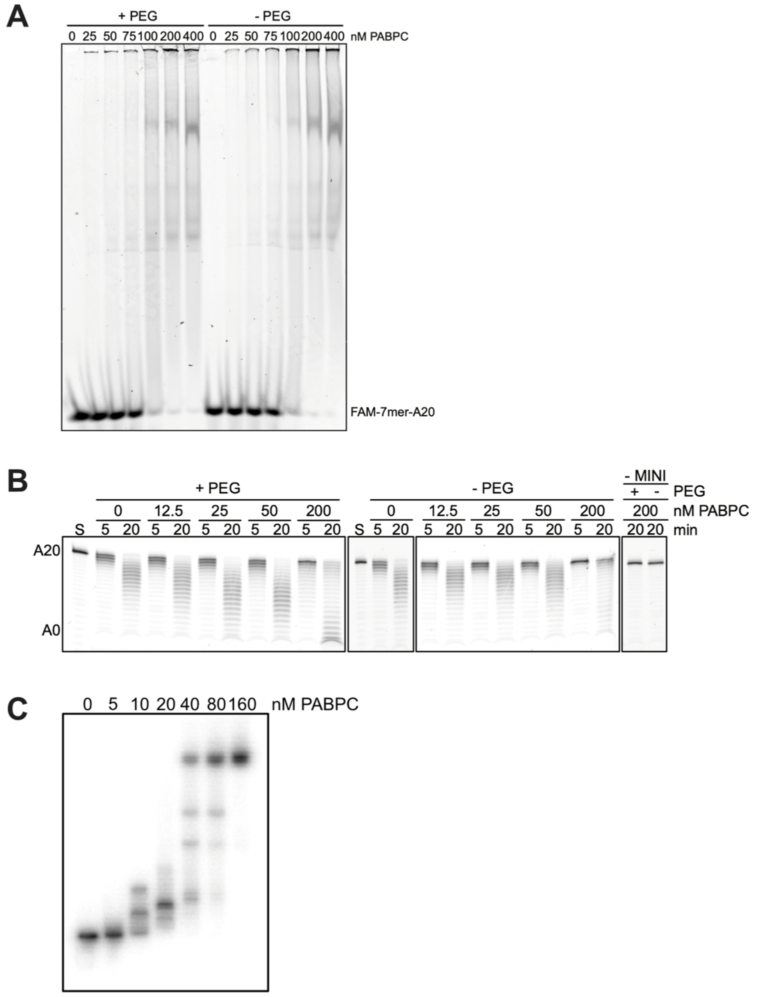

Supplemental Figure 4. PABPC modestly stimulates basal deadenylation

(A) Binding of PABPC to the FAM-7mer-A_{20} RNA. Indicated concentrations of PABPC were incubated with 25 nM FAM 7mer-A_{20} RNA at 25 °C for 20 minutes in the presence of tRNA and in the presence or absence of PEG. Then, RNA-protein complexes were separated by electrophoresis on a nondenaturing polyacrylamide gel run at 200 V and room temperature.

(B) PABPC stimulates deadenylation of FAM 7mer. FAM 7mer-A_{20} RNA (25 nM) was incubated with the indicated concentrations of PABPC in the presence or absence of PEG. In these assays, tRNA was present as in the binding assay (panel A). The deadenylation reaction was started with ^{Dm}CCR4-NOT_{MINI} (1 nM) and allowed to proceed for 5 or 20 min as indicated.

(C) Binding of PABPC to SRE^{WT}only-A_{70} RNA. SRE^{WT}only-A_{70} RNA (5 nM) was incubated with the indicated concentrations of PABPC, and RNA-protein complexes were analyzed as in (A). Reactions contained neither tRNA nor PEG. The simplest interpretation of the retarded bands is that up to four molecules of PABPC can be accommodated on one RNA molecule. With an A_{70} tail and one molecule of PABPC covering ~30 nt (Baer and Kornberg, 1980; Sachs et al., 1987; Schäfer et al., 2019), binding of two to at most three molecules would be expected. Thus, PABPC binding can extend into the RNA body. The complex pattern seen at 10 nM PABPC was reproducible but has not been further investigated. The affinity of PABPC for an isolated poly(A) binding site is in the low nanomolar range (Sachs et al., 1987; Görlach et al., 1994; Kühn and Pieler, 1996). Thus, at 5 nM RNA and 10 nM PABPC, binding is not expected to be complete in spite of excess protein over RNA.

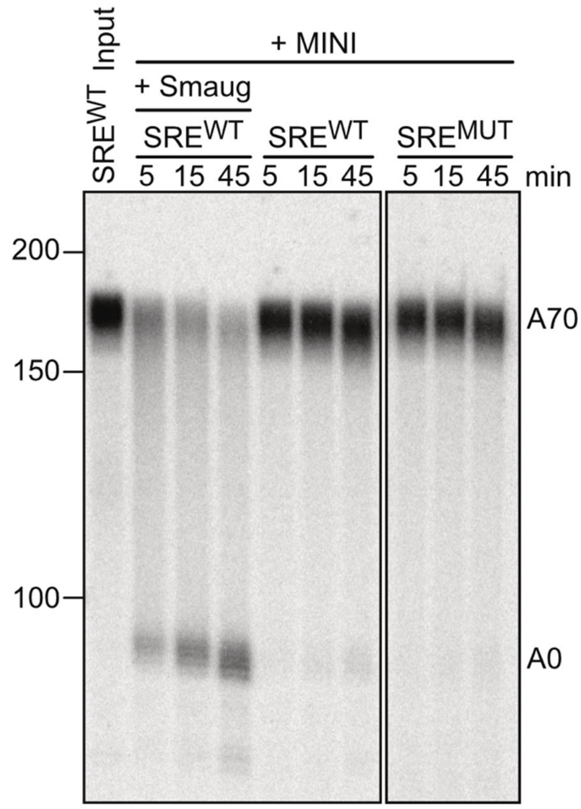

Supplemental Figure 5. Smaug-independent deadenylation of a long substrate RNA is distributive

20 nM SRE^{WT}only-A_{70} RNA was preincubated for 20 min with 80 nM Smaug or buffer. SRE^{MUT}only-A_{70} RNA was preincubated with buffer. Deadenylation was then initiated by the addition of 5 nM ^{Dm}CCR4-NOT$_{MINI,}$ and aliquots were withdrawn at the time points indicated. Co-existence of full-length RNA and completely deadenylated product in the Smaug-containing reaction confirms processive activity, as in Fig. 6B. In the absence of Smaug, modest shortening of the entire population of substrate RNA, mostly visible at the latest time point, indicates weak, distributive activity. In this reaction, very small amounts of completely deadenylated product are also visible. These are no longer present when an SRE^{MUT} substrate is used, suggesting the possibility that the CCR4-NOT preparation is contaminated by small amounts of a Smaug-like protein.

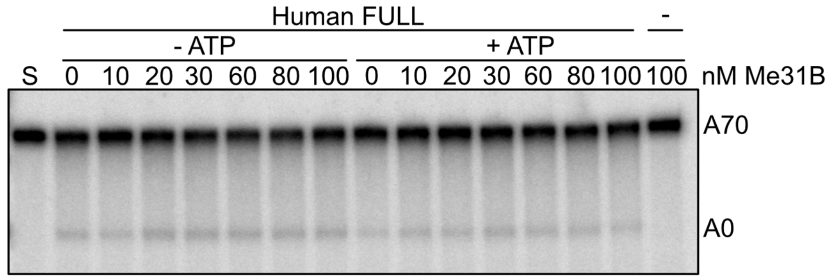

Supplemental Fig. 6. Me31B does not stimulate deadenylation

Radiolabeled SRE^{MUT}-A_{70} RNAs (10 nM) was preincubated with the indicated concentrations of Me31B before deadenylation was started by the addition of 10 nM ^{Hs}CCR4-NOT$_{FULL}$. Reactions were stopped after 40 min and analyzed by denaturing gel electrophoresis. A control reaction (last lane) was incubated with Me31B only (100 nM). ATP was present at 0.8 mM where indicated. Similar assays were carried out with the *Drosophila* CCR4-NOT complex and did not reveal any stimulation of deadenylation by Me31B either.

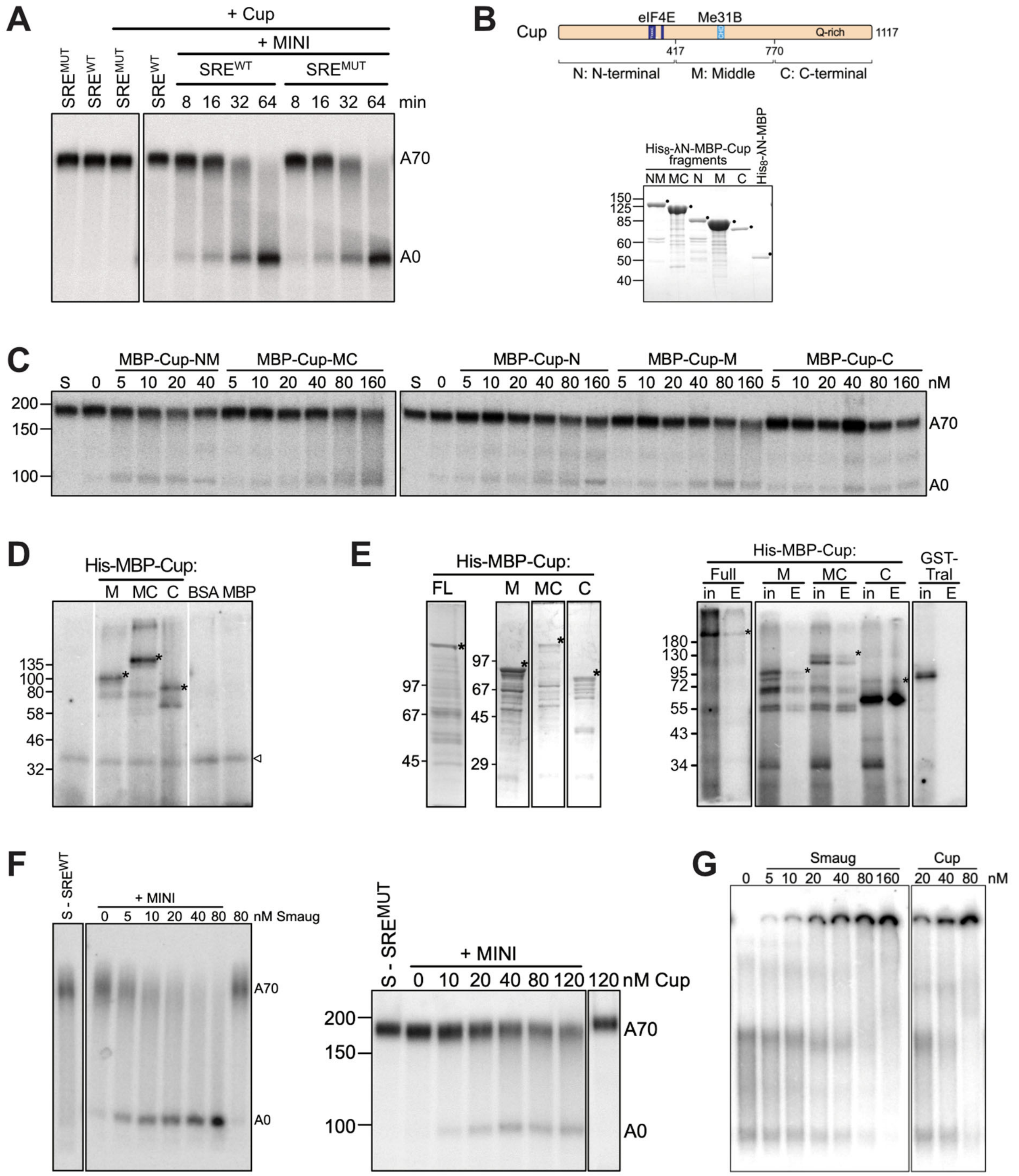

Supplemental Figure 7. Cup contributes to deadenylation

(A) Stimulation of deadenylation by Cup is independent of SREs. Radiolabeled SRE^{WT}-A_{70} or SRE^{MUT}-A_{70} RNA (20 nM) was preincubated with 80 nM Cup before deadenylation was started by the addition of 2 nM ^{Dm}CCR4-NOT$_{MINI}$. Aliquots were taken at the times indicated and analyzed by denaturing gel electrophoresis. The first two lanes show RNA incubated without proteins, the subsequent two lanes contain RNA incubated with Cup only.

Supplemental Figure 7 (continued). Cup contributes to deadenylation
(B) A scheme of Cup and its separation into three fragments is shown at the top. The bottom presents an SDS-polyacrylamide gel with the purified His_8-λN-MBP-tagged Cup fragments and the His_8-λN-MBP tag without Cup. These protein preparations were used for the experiment shown in **Fig. 7B**. Desired proteins are labeled with black dots.
(C) The ability of Cup to stimulate deadenylation is distributed over the protein. 5 nM ^{32}P-SRE^{MUT}only-A_{70} RNA was preincubated in the presence of varying concentrations of different Cup fragments or in their absence, as indicated. Deadenylation was started by the addition of 1 nM ^{Dm}CCR4-NOT$_{MINI}$. Reactions were allowed to proceed for 30 min. S, unreacted substrate. Controls in a separate experiment showed that the Cup fragments were devoid of nuclease activity, i. e. deadenylation was CNOT-dependent. A representative experiment of n=3 is shown.
(D) Control proteins MBP and BSA are not UV cross-linked to RNA. His-MBP-tagged Cup fragments lacking λN as well as similar amounts of the control proteins BSA and MBP were UV-cross-linked to radiolabeled RNA. Cross-linking products were analyzed by SDS-polyacrylamide gel electrophoresis and autoradiography. Bands corresponding to the calculated molecular weights of Cup fragments are labeled with asterisks. Cross-linking products of BSA or MBP would have been expected at ~66 or ~43 kDa, respectively. The identity of the band marked with an arrowhead is unknown, but it was also present in a reaction without proteins (first lane).
(E) Cross-linked proteins were identified as fragments of Cup by affinity purification. Left panel, Coomassie-stained SDS-polyacrylamide gel lanes showing His-MBP-tagged Cup and Cup fragments lacking λN.. Bands corresponding to the desired proteins are marked. Right panel: Proteins shown in the left panel were UV-cross-linked to RNA (in, input fraction; 30 % of total reaction). 70% of each reaction were incubated with Ni-NTA matrix in the presence of 6M urea and 0.05 % NP-40, and bound protein-RNA complexes were eluted (E, eluate). Input and eluate were analyzed by SDS-polyacrylamide gel electrophoresis and autoradiography. Bands corresponding to the proteins of interest are labeled. In a control reaction, GST-Tral, lacking a His tag, was cross-linked to RNA and passed over a Ni-NTA column. The protein was not retained on the column.
(F) Titration of Smaug (left panel) and Cup (right panel) in deadenylation. 20 nM SREonly-A_{70} RNA, wild-type or mutant as indicated, was preincubated for 30 min with the indicated concentrations of Cup or Smaug, respectively, then deadenylation was started by the addition of 2 nM ^{Dm}CCR4-NOT$_{MINI}$ and stopped after 40 min. PEG was present in the Smaug titration, but not in the Cup titration.
(G) Titration of Smaug and Cup in RNA binding. 10 nM SRE^{WT}only-A70 RNA was incubated at 25 °C for 20 minutes with the indicated concentrations of Smaug or Cup in the absence of PEG. Then, RNA-protein complexes were separated by electrophoresis on a non-denaturing polyacrylamide gel. Retarded RNA-protein complexes were stuck in the wells.

Supplemental Table 1. Accession numbers of sequences amplified for expression constructs

Protein	Isoform	FlyBase ID
NOT1	PC	FBpp0111584
NOT1	PE	FBpp0111586
NOT2	PA	FBpp0078390
NOT3	PA	FBpp0085398
CAF40	PA	FBpp0074513
CCR4	PA	FBpp0083951
CAF1	PA	FBpp0075790
NOT10	PA	FBpp0082112
NOT11	PB	FBpp0072098
Smaug	PA	FBpp0076277
Cup	PB	FBpp0288761
PABPC	PA	FBpp0085917
Me31B	PB	FBpp0079566
Trailer hitch	PA	FBpp0075691
Belle	PB	FBpp0306433
eIF4E-1	PA	FBpp0076218

Supplemental Table 2. Sequences of synthetic oligonucleotides
Capital letters indicate the start of the corresponding gene. Point mutations are indicated with bold letters.

Gene etc.	Forward sequence (5'-3')	Reverse sequence (5'-3')	Plasmid
NOT1 PC	gctctagaATGGCTAGTAACGTAGAGAGCCAACTG	TGGCTAATAGCACGCGCTATC	pFBDM-His-NOT2_NOT1(Full)
NOT1 PE	gctctagaATGGCTAGTGACACATCTTGGATTAATC	TGGCTAATAGCACGCGCTATC	pFBDM-His-NOT2_NOT1(PE)
NOT1 MINI	ggactagtatgcatcatcaccatcaccatcaccatGTGACTGTGCCACCAGAG	cccaagcttTCAGTTGATGGTGGCTAC	pET28a-MBP-His8-NOT1(MINI)
			pFBDM-His8-NOT2_His8-NOT1(MINI)
NOT2	tcccccgggatgcatcatcaccatcaccatcaccatATGGCGAATTTAAATTTTC	ggggtaccTTATACAGACTGTCCATTC	pFBDM-His8-NOT2_His8-NOT1(MINI)
NOT3	tcccccgggatgcatcatcaccatcaccatcaccatATGGCTGCGACGAGAAAATTG	ggggtaccTCAATTCAGCTCCTTGTC	pFBDM-CAF40-FLAG_His8-NOT3
Caf40	ggaattcATGAGTGCTCAACCGAGTC	cccaagcttctacttatcgtcgtcatccttgtaatcGGAGCCCAGTGGCGACATG	pFBDM-CAF40-FLAG_His8-NOT3
CAF1	gcgcggatccatgtctcatcatcatcatcatcatcaccacATCAAATGGACAATGCCC	gctctagaTCATGAAGCGCTGTTCGTC	pFBDM-CCR4-FLAG_His8-CAF1
CCR4	ggatctcgagATGAAAGGCAATCATTATAAA	tcccggtaccttaatccttgtcatcgtcgtccttgtagtcGGCCCGGCGATTGATCAGC	pFBDM-CCR4-FLAG_His8-CAF1
CAF1 (MUT)	CACTATGTGGCCATGG**c**CACCG**c**GTTTCCAGGCGTGGTAG	CTACCACGCCTGGAAAC**g**CGGTG**g**CCATGGCCACATAGTG	pFBDM-CCR4-FLAG_His8-CAF1(MUT)
			pFBDM-CCR4-FLAG(MUT)_His8-CAF1(MUT)
CCR4 (MUT)	GCTGCTGCTGTGCGGTG**c**CTTC**gc**CTCGCTACCCGATTCAG	CTGAATCGGGTAGCGAG**gc**GAAG**g**CACCGCACAGCAGCAGC	pFBDM-CCR4-FLAG(MUT)_His8-CAF1
			pFBDM-CCR4-FLAG(MUT)_His8-CAF1(MUT)
Smaug	ctagctagcATGAAGTACGCAACTGGAAC	ataagaatgcggccgcctatttatcatcatcatctttataatcGAATAGCGTAAAATGTTG	pFBDM-Smaug-FLAG
Cup	tcccccgggctttattttcagggc	ccgctcgagttacttatcgtcgtcatccttgtaatcATGAAACTCATCCCCGC	pFBDM-Cup-FLAG
Tral	cggaattcATGAGCGGGGGATTACCG	atagccatggtcagtggtgatgatgatgatgatgatgTTGTGAAACTGCCGCCAC	pFBDM-Tral-His8

PABPC	cgcggatccatgcatcatcatcatcatcatcaccacATGGCTTCTCTATACGTC	ccgctcgagTTAGTTGGCGGGCTCGGTG	pET28a-PABPC
Belle	tgcagtctcgagatgcatcatcatcatcatcatcaccacAGTAATGCTATTAACC	gtcgacaagcttTCATTGAGCCCACCA	pFastBac1-His8-Belle
CupNM	ccgctcgagATGCAAATGGCCGAAGC	cgcctaggTTATCGACGCCATTTG	pnEK-His8-MBP-CupNM
CupMC	ccgctcgagGACGAGTCCATC	ccgcctaggTTAATGAAACTCATCC	pnEK-His8-MBP-CupMC
CupN	ccgctcgagATGCAAATGGCCGAAGC	cgcctaggTTAGTCACTGATTAGGTTC	pnEK-His8-MBP-CupN
CupM	ccgctcgagGACGAGTCCATC	cgcctaggTTATCGACGCCATTTG	pnEK-His8-MBP-CupM
CupC	ccgctcgagCGAAACTCACTGAAC	ccgcctaggTTAATGAAACTCATCC	pnEK-His8-MBP-CupC
SmaugNM	cgggatccATGAAGTACGCAACTGGAAC	tcccccgggTTAAATATTGGCCCGTTCCTTC	pET28a-SmaugNM
SmaugMC	cgggatccTGCCCCGCAAGCGGCAG	tcccccgggTTAGAATAGCGTAAAATGTTG	pGEX6p1-SmaugMC
SmaugN	cgggatccATGAAGTACGCAACTGGAAC	tcccccgggTTACAACGAGGATGAGGCCAC	pGEX6p1-SmaugN
SmaugM	atatggtctcatggtAATTATATTAAGTTCCACACGCGC	tatctcgagttaATTATTCAGCGACCGGC	pET-SUMOadapt-SmaugM
SmaugC	cggaattcCTTAACCGGGTAGAACAAG	tcccccgggTTAGAATAGCGTAAAATGTTG	pGEX6p1-SmaugC
His-tag	catgggcagcagccatcatcaccatcaccatcaccattc	catggaatggtgatggtgatggtgatggctgctgcc	pnEK-His8-MBP
2xBoxB	gatccGGGCCCTGAAGAAGGGCCCATATAGGGCCCTGAAGAAGGGCCCt (BamHI fragment)	ctagaGGGCCCTTCTTCAGGGCCCTATATGGGCCCTTCTTCAGGGCCCg (XbaI fragment)	pBSK-nLuc-2xBoxB
BRE^{WT}	ggaagatctGAATTCGCTTAGTTTTAATATG	gctctagaggatccTTAAATCTAACATAGAAC	pBSK-nLuc-BRE^{WT}
BRE^{MUT} EcoRI	AATTCGCTTAGTTTTAAT**ta**GTTTT**ta**AT**tg**AG**at**TGTTCTCTGTCTTTGTT**at**TTTA**g**AT**t**TTCGTGCACTT (EcoRI fragment1)	AA**a**AT**c**TAAA**at**AACAAAGACAGAGAACA**at**CT**ca**AT**ta**AAAAC**ta**ATTAAAACTAAGCG (EcoRI fragment2)	pBSK-nLuc-BRE^{MUT}
BRE^{MUT} BamHI	GTCCTAGTCCATTATT**t**TA**g**ATTATT**t**TG**g**GTTTTG**gt**TTCT**ta**GTTAGATTTAAG (BamHI fragment1)	GATCCTTAAATCTAAC**ta**AGAA**ac**CAAAAC**c**CA**a**AATAAT**c**TA**a**AATAATGGACTAGGACAAGTGCACG (BamHI fragment2)	

Supplemental Table 3. ReLo and Y2H DNA constructs used in this study.
All bait and prey sequences were from *Drosophila melanogaster*.
The split-ubiquitin Y2H cloning vectors pDHB1-MJ (JK16) and pPR3-N-MJ (JK18) were generated by introducing the blunt end restriction sites Eco47III and SmaI into the multiple cloning sites of pDHB1 and pPR3-N (Jeske et al., 2015), respectively. The plasmids pAc5.1-EGFP (T5-MJ), pAc5.1-mCherry (T7-MJ), pAc5.1-PH-mCherry-CAF1 (HK96), pAc5.1-PH-mCherry-CAF40 (HK97), pAc5.1-PH-mCherry-NOT2 (HK99), pAc5.1-PH-mCherry-NOT3 (HK100), pAc5.1-CCR4-mCherry-PH (EB7), and pAc5.1-NOT1-mCherry-PH (EB5) have been described previously (Salgania et al., 2022). Generation of the DNA constructs listed in the table was performed according to the cloning strategy described previously (Salgania et al., 2022).

**Vector** (insertion site) (code)	**Final DNA construct**	**DNA template information**	**Code**
**pAc5.1-EGFP** (EcoRV) (T5-MJ)	pAc5.1-EGFP-**Smaug**	*smaug* cDNA	F31-MJ
**pAc5.1-mCherry** (EcoRV) (T7-MJ)	pAc5.1-mCherry-**Smaug**	*smaug* cDNA	H28-MJ
**pAc5.1-PH-mEGFP** (FspAI) (JM50)	pAc5.1-PH-mEGFP-**NOT3 1-241**	pFL-Flag-NOT3	JM69
	pAc5.1-PH-mEGFP-**NOT3 242-686**	pFL-Flag-NOT3	JM70
	pAc5.1-PH-mEGFP-**NOT3 687-844**	pFL-Flag-NOT3	JM71
**pDHB1-MJ** (Eco47III) (JK16)	pDHB1-MJ-**Smaug**	*smaug* cDNA	JK46
**pPR3-N-MJ** (SmaI) (JK18)	pPR3-N-MJ-**CAF1**	pMTV5-Myc-CAF1 (Temme et al., 2010)	JK76
	pPR3-N-MJ-**CAF40**	pET19-CAF40	JK77
	pPR3-N-MJ-**CCR4**	pMTV5-Myc-CCR4	JK78
	pPR3-N-MJ-**NOT2**	pSPL_Strep_NOT1_NOT2	JK88
	pPR3-N-MJ-**NOT3**	pFL-Flag-NOT3	JK89
	pPR3-N-MJ-**NOT1 1-751**	pSPL_Strep_NOT1_NOT2	JK84
	pPR3-N-MJ-**NOT1 752-910**	pSPL_Strep_NOT1_NOT2	FH16
	pPR3-N-MJ-**NOT1 911-1092**	pSPL_Strep_NOT1_NOT2	FH13
	pPR3-N-MJ-**NOT1 1093-1687**	pSPL_Strep_NOT1_NOT2	JK86
	pPR3-N-MJ-**NOT1 1688-1964**	pSPL_Strep_NOT1_NOT2	FH4
	pPR3-N-MJ-**NOT1 1965-2480**	pSPL_Strep_NOT1_NOT2	JK87
	pPR3-N-MJ-**NOT3 1-241**	pFL-Flag-NOT3	MS5
	pPR3-N-MJ-**NOT3 242-686**	pFL-Flag-NOT3	MS9
	pPR3-N-MJ-**NOT3 687-844**	pFL-Flag-NOT3	MS13

REFERENCES

Baer B. W., and Kornberg R. D. (1980). Repeating structure of cytoplasmic poly(A)-ribonucleoprotein. Proc Natl Acad Sci U S A. 77,1890-1892.

Görlach, M., Burd, C. G., and Dreyfuss, G. (1994). The mRNA poly(A)-binding protein: localization, abundance, and RNA-binding specificity. Exp Cell Res *211*, 400-407.

Jeske, M., Bordi, M., Glatt, S., Muller, S., Rybin, V., Muller, C.W., and Ephrussi, A. (2015). The Crystal Structure of the Drosophila Germline Inducer Oskar Identifies Two Domains with Distinct Vasa Helicase- and RNA-Binding Activities. Cell Rep *12*, 587-598.

Kühn, U. and Pieler, T. (1996). Xenopus poly(A) binding protein: Functional domains in RNA binding and protein – protein interaction. J Mol Biol *256*, 20-30.

Sachs A. B., Davis R. W., Kornberg R. D. (1987). A single domain of yeast poly(A)-binding protein is necessary and sufficient for RNA binding and cell viability. Mol Cell Biol 7, 3268-3276.

Schäfer, I. B., Yamashita, M., Schuller, J. M., Schüssler, S., Reichelt, P., Strauss, M., and Conti, E. (2019). Molecular Basis for poly(A) RNP architecture and recognition by the Pan2-Pan3 deadenylase. Cell 177, 1619-1631.

Salgania, H.K., Metz, J., and Jeske, M. (2022). ReLo: a simple colocalization assay to identify and characterize physical protein-protein interactions. BioRxiv, doi: 10.1101/2022.1103.1104.482790

Temme, C., Zhang, L.B., Kremmer, E., Ihling, C., Chartier, A., Sinz, A., Simonelig, M., and Wahle, E. (2010). Subunits of the Drosophila CCR4-NOT complex and their roles in mRNA deadenylation. RNA *16*, 1356-1370.
